# Water-Driven Cascade Specific Detection of Al(III)/Hg(II) and CN^–^/GSH in Real Water Samples

**DOI:** 10.1021/acsomega.5c04268

**Published:** 2025-07-14

**Authors:** Rabia Ardahanlı, Abdullah S. Hussein, Ferruh Lafzi, Sinan Bayindir, Haydar Kilic

**Affiliations:** † Department of Chemistry, Faculty of Sciences, 37503 Ataturk University, 25240 Erzurum, Turkey; ‡ Department of Chemistry, Faculty of Sciences and Arts, 162312 Bingol University, 12000 Bingol, Turkey; § College of Education Chemistry Department, Salahaddin University-Erbil, 44001 Erbil, Iraq

## Abstract

This work presents novel rhodamine hybrids with *para*-quinone methide groups as ion-sensing probes. The probes, **P1** and **P2**, exist in both open and closed ring conformations, making them promising candidates for sensors due to their distinct absorption and fluorescence properties. Fluorescence studies demonstrated that **P1** acts as a “turn-on” sensor for Hg­(II) in water, while **P2** responds similarly to Al­(III) in water–methanol solutions. The detection limits (LODs) were determined to be 389 nM for Hg­(II) and 857 nM for Al­(III). In addition, the probes exhibit “turn-off” responses to cyanide and glutathione in the presence of Al­(III) and Hg­(II), with LODs of 680 nM and 1.21 μM, respectively. By visually inspecting colorimetric changes in filter paper and water samples, we also looked into the feasibility of these probes in real-world settings. Overall, this work demonstrates the suitability of probes for the detection of mercury and aluminum in water samples from natural environments.

## Graphical abstract

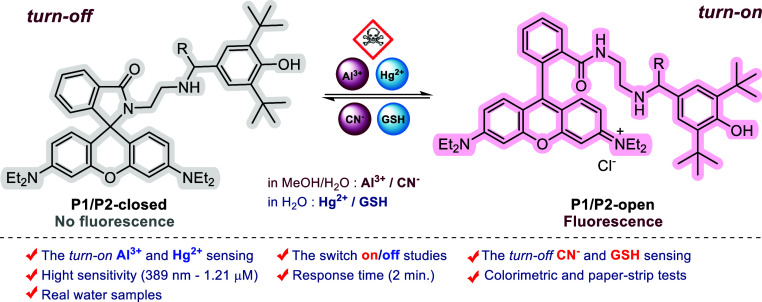

## 1 Introduction

In recent years, there has been a growing interest in fluorescent organic materials (FOMs) in both sensor and conductive technologies.^1–4^ In ion detection, simpler and more sensitive techniques, such as colorimetry and fluorometry, are emphasized, providing an inexpensive alternative to other traditional techniques.^5–7^ This calls for the creation of selective organic compounds that would detect ions in complex mixtures.^8–11^ One such compound of interest, with increasing attention in today’s research, is rhodamine (Rh) and aryl *para*-quinone methide (*p*QM).^12,13^ The development of Rh-based organic derivatives has become a significant area of modern research due to their versatility in fields such as chemical and biological sensing, organic light-emitting diodes (OLEDs), and organic photovoltaics (OPVs).^14–17^ The ability to detect mercury and aluminum in this research has successfully achieved international detection levels for these elements. Mercury (Hg­(II)), a highly toxic heavy metal, is an increasing environmental concern due to its industrial use.^18,19^ Stringent regulations limit mercury levels in drinking water to below 2 ppb to prevent health risks such as kidney damage.^20,21^ Current detection methods for mercury and other heavy metals often require costly, complex instruments that can be limited in sensitivity and selectivity, such as atomic absorption and emission spectroscopy.^22^ As a result, there is a critical need for simpler, more efficient detection methods to safeguard public health.^23^ On the other hand, aluminum (Al­(III)), a lightweight, silvery metal, is abundant in the Earth’s crust and possesses valuable properties such as corrosion resistance, low density, and malleability.^24,25^ These characteristics make it indispensable in the aerospace, automotive, packaging, and construction industries.^26,27^ However, excessive aluminum exposure can pose health risks, including neurological disorders such as Alzheimer’s and bone diseases, kidney issues, and respiratory problems.^28^ To mitigate these risks, managing aluminum exposure carefully, including proper handling and disposal to reduce environmental contamination and ensure the safety of human health, is crucial.^29^ The World Health Organization (WHO) recommends an average daily aluminum intake of 3–10 mg, and the permissible limit for aluminum in drinking water is set at 5 mg/L (or 7.41 μM).^24,30,31^ These guidelines are designed to ensure the safety of drinking water and protect public health from harmful effects of aluminum. In contrast, cyanide is extremely toxic and can cause rapid death by affecting vital systems, necessitating much stricter control.^32,33^ Although regulatory limits for cyanide in drinking water are set at very low levels (1–2 μM), even small exposures can be fatal.^30,34^ Consequently, precise methods for detecting and eliminating this pollutant are crucial. Although cyanide forms less toxic metal complexes utilized in specific industrial applications, organic-based binder research may be a more productive means of detection and elimination.^35,36^ Glutathione (GSH), an important cellular antioxidant, protects against various diseases.^37^ Although GSH levels can vary significantly depending on the cell type, they generally range from approximately 0.5 to 10 mM in healthy tissues and can reach up to 15 mM in certain cancerous tissues.^38^ That is, abnormal GSH concentrations have also been linked to conditions such as liver injury and Alzheimer’s disease.^39,40^ Therefore, an accurate glutathione determination is crucial. Rhodamines are compounds with multifunctionality that are characterized by both light-emitting (fluorophore) and semiconducting activity. In this study, we have synthesized two novel Rhs modified with *para*-quinone methide groups and examined their ability to detect ions with colorimetric and spectroscopic analysis.

## 2 Results and Discussions

### 2.1 Synthesis

Rhodamine-based chemical compounds have potential technical applications. In this study, two new rhodamine hybrids, **P1** and **P2**, containing *para*-quinone methide units, were synthesized by using a multistep reaction method, and their sensor properties were investigated. One of the precursors, the spirolactam intermediate Rh–NH_2_, was synthesized by reacting rhodamine B (**1**) and ethylenediamine in ethanol (Scheme S1).^41^ Additionally, *para*-quinone methide derivatives were synthesized using standard procedures as stated in the literature.^42^ The N–H alkylation of Rh by *p*QMs was then carried out in hexafluoroisopropanol at room temperature for 12 h in the absence of a catalyst, and the probes **P1** and **P2** were formed in yields of 79% and 31%, respectively (Scheme 1).

**Scheme 1:**
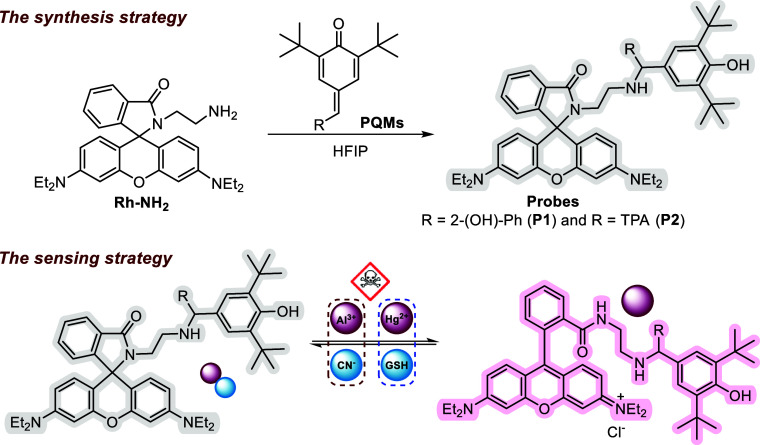
Synthesis and Sensing Strategies of Probes **P1** and **P2**

### 2.2 Recognition Properties

Following the synthesis of probes **P1** and **P2**, we employed UV–vis and fluorescence spectroscopy to investigate the ion-sensing capabilities. A range of solvents, both organic (MeOH, EtOH, DMSO, THF) and aqueous, were tested (Figures S5–S7). The probes were exposed to a diverse range of cations—Ag^+^, Al^3+^, Ba^2+^, Ca^2+^, Cd^2+^, Co^2+^, Cu^2+^, Fe^2+^, Fe^3+^, Hg^2+^, K^+^, Mg^2+^, Mn^2+^, Na^+^, Ni^2+^, Pb^2+^, Sb^3+^, and Zn^2+^ (all as their chloride salts)—and anions—[Bu_4_N]­F, [Bu_4_N]­Cl, [Bu_4_N]­Br, [Bu_4_N]­I, [Bu_4_N]­AcO, [Bu_4_N]­HSO_4_, [Bu_4_N]­ClO_4_, [Bu_4_N]­CN, [Bu_4_N]­SCN, [Bu_4_N]­H_2_PO_4_, and [Bu_4_N]­OH. Initial experiments revealed that the probes **P1** and **P2** exhibited specifically selective responses to Al­(III) ions in the MeOH/H_2_O (v/v: from 10/0 to 9/1) mixture (Figures S5D, S6, and S7D). On the other hand, **P1** demonstrated interactions with Hg­(II) ions in water solutions, including tap water (Figure S6C,D). The rhodamine ring’s conformational state is pH-sensitive, adopting an open form in acidic conditions and a closed form in basic environments (Figures S6F and S8). On the other hand, for each selected measurement, a fluorescence response could be obtained within 2 min, while distinct color changes could take up to about 2 to 5 min. Therefore, studies were conducted at a physiological pH of 7, both with and without the addition of HEPES buffer, within a 2 min time frame. Following optimization experiments, UV–vis and fluorescence spectroscopic studies of probes were conducted in HEPES/MeOH (v/v:1/1) and HEPES buffer. Accordingly, through analysis of the UV–vis spectra of probes **P1** and **P2**, distinct peaks of π–π interactions appeared at 273/314/381 nm for **P1** and at 302/450 nm for **P2** (Figures 1A, 1A’, and S9A). The interaction of rhodamine rings with ions, especially Al­(III) and Hg­(II), resulted in ring opening with the formation of an overwhelming absorbance band in the range of about 560 nm, along with a distinct color change (Figure 1A). When analyzed in MeOH/H_2_O (v/v:1/1)/H_2_O, in the presence of Al­(III)/Hg­(II) from respective ions, novel peaks were present at approximately 560 nm for **P1**, along with a color switch from colorless to dark pink. Remarkably, a depletion of the 381 nm peak is observed in the presence of aluminum. In contrast, the absence of new peaks at around 560 nm in the case of an interaction between aluminum and probe **P2** indicates that the ring remains in its closed form. However, with this metal ion, merely the 450 nm peak selectively disappears in intensity (Figure S9A). Remarkable to note is that there is absolutely no gross change with other metal ions or general anions apart from specific interactions described earlier. In addition to UV–vis spectroscopy, fluorescence spectroscopy was employed to gain deeper insights into the interaction of probes **P1** and **P2** with various metal ions. We observed that upon excitation at 500 nm, probe **P1** displays specific behavior in response to Hg­(II) in H_2_O and Al­(III) in MeOH/H_2_O (v/v:1/1), causing immense emission at 582 nm (Figures 1B, 1B’, and S9B). This proves that probe **P1** is capable of acting as a selective turn-on fluorescent sensor for such cations. Correspondingly, **P2**, excited at 450 nm in MeOH/H_2_O, exhibited a pronounced fluorescent peak at 580 nm upon the addition of Al­(III) ions (Figures 1B, 1B’, and S9B). The results of this fluorescence agree with our UV–vis spectroscopy results, which demonstrate selective ion sensing by these probes.

**Figure 1:**
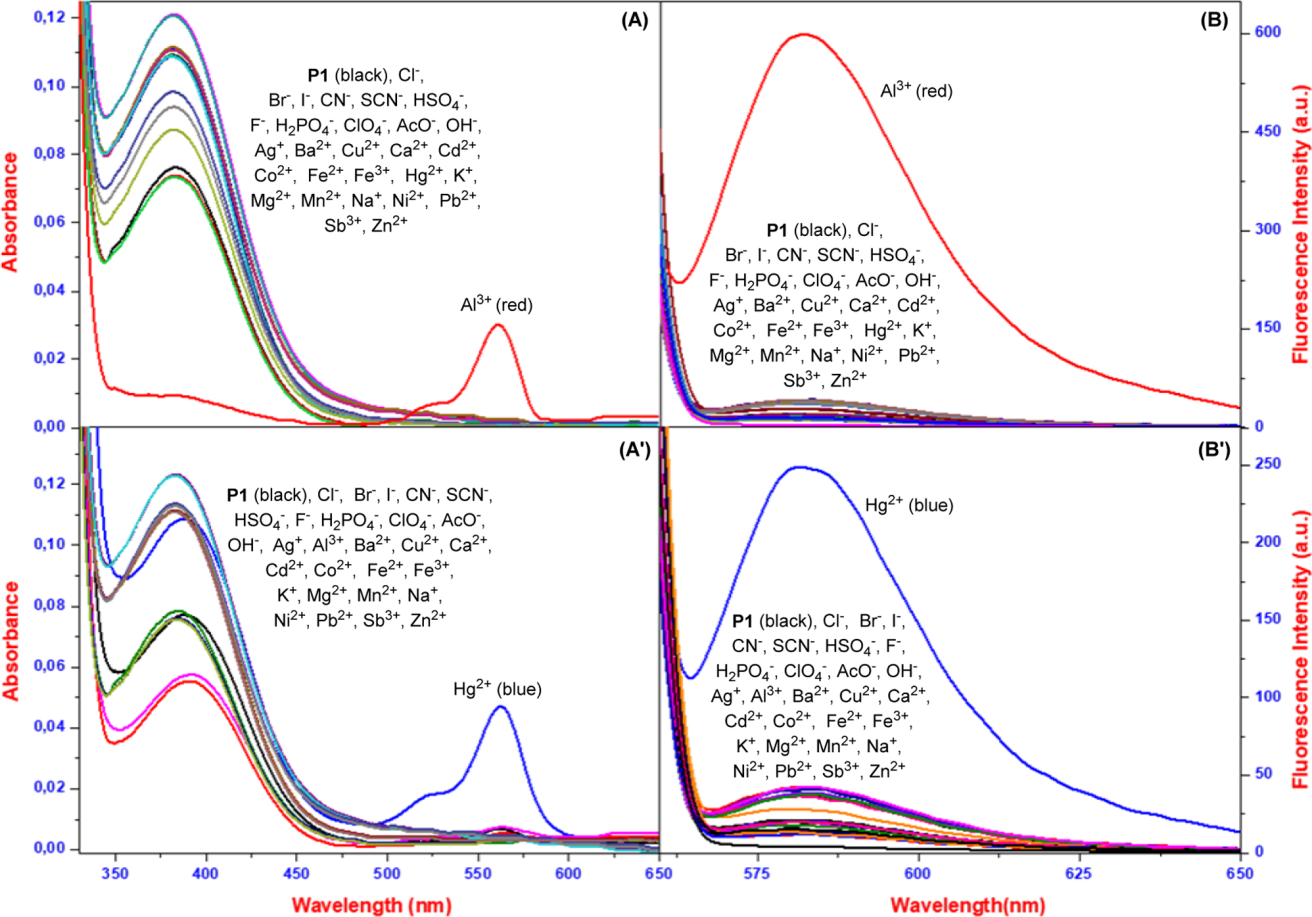
UV–vis (A,A′) and fluorescence (B,B′) spectra of **P1** with the absence and presence of ions in MeOH/H_2_O (A,B) and water (A′**,**B′) at pH 7.

Moreover, to test their applicability in actual scenarios, we examined their selectivity in the presence of other cations in Figures 2 and S10. In aqueous media for the detection of Hg­(II), **P1** is highly selective and is not affected by interference from other cations. Remarkably, the addition of glutathione (GSH) lowered the **P1**–Hg­(II) complex intensity within 1 min, which makes it a promising candidate to be used as a sensor for GSH (Figures 2B and S10B). Upon detection of Hg­(II) in water, in a binary solvent system of MeOH/H_2_O (v/v:1/1), both of the complexes, namely, **P1**+Al­(III) and **P2**+Al­(III), are highly selective in their response. Only cyanide anions are capable of decreasing their intensity significantly (Figures 2A, S10A,C). This surprising observation makes these probes, Al­(III) complexes, good turn-off fluorescent sensors for the detection of cyanide anions in MeOH/H_2_O (v/v:1/1) within a 1 min time frame. Moreover, another essential aspect of the common ion study involved testing with mixtures of metal ions. It was found that the presence of other ions did not interfere with the solvent-selective detection of Hg­(II)/Al­(III) ions, and consistent results were obtained across the tests (Figure S10E,F).

**Figure 2:**
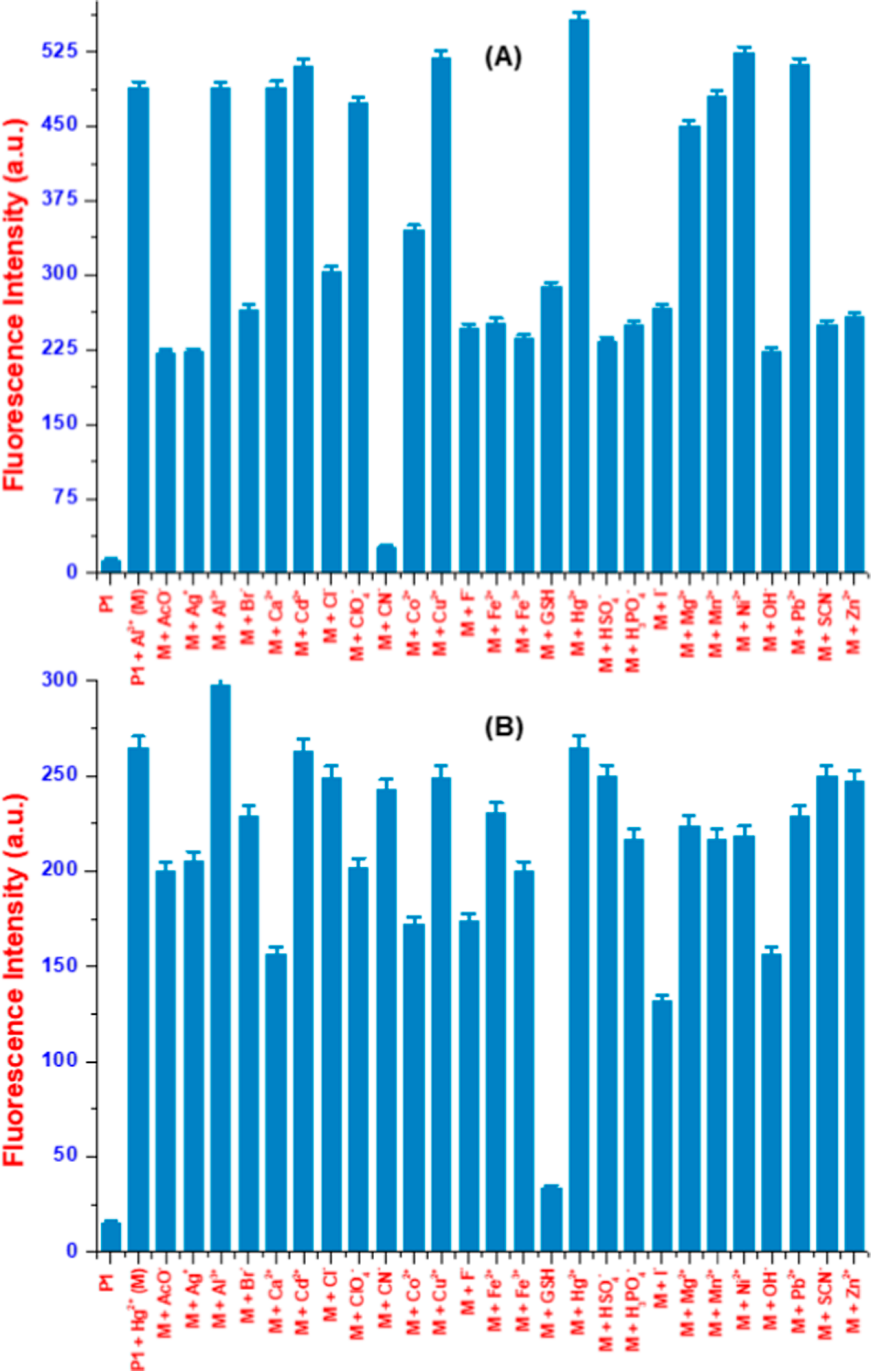
Fluorescence bar graphics of **P1**–Al­(III) (A) and **P1**–Hg­(II) (B) upon adding different ions up to 1 equiv in MeOH/H_2_O (for Al­(III)) and water (for Hg­(II)) at pH 7.

Following general spectral studies, fluorescence titrations were performed to examine the interaction between **P1** and **P2** molecules with mercury and aluminum ions (Figure S11A–C). As the concentration of metal ions increased, the fluorescence intensity of both probes gradually rose, reaching a plateau at 5 μM for aluminum (Figure S11A) and 4 μM for mercury (Figure S11B). The limit of detection (LOD) and limit of quantification (LOQ) were determined using eqs 1 and 2 (details in the Experimental Section). The calculated LOD/LOQ values were 389 nM/1.18 μM for MI–Hg­(II) and 857 nM/2.60 μM for **P1**–Al­(III) (Figures S11A′,B′). Additionally, LOD and LOQ values for **P2**–Al­(III) were determined to be 1.23 and 3.72 μM, respectively (Figure S11C). Overall, LOD values from **P1** and **P2** enable sensitive detection of Al­(III) ions in aqueous solutions at the nanomolar and micromolar levels. The reversible binding of **P1**–Hg­(II) and **P1**–Al­(III) complexes with GSH and cyanide ions suggested their potential as sensors for these analytes (Figure S12A,B). Fluorescence titration experiments with increasing GSH and cyanide ion concentrations confirmed this hypothesis. The fluorescence intensity at 580 nm decreased proportionally with increasing GSH and CN^–^ ion concentrations, reaching a plateau at around 4.0 μM (Figure S12A) and 5.0 μM (Figure S12B), respectively. The calculated LOD/LOQ values were 1.21/3.67 μM for GSH and 0.680/2.06 μM for CN^–^ (Figure S12A,B). These results highlight these complexes’ high sensitivity and accuracy for detecting GSH and cyanide. To evaluate the potential of these probes as sensors, we determined their binding affinity for Hg­(II) and Al­(III) ions using Job’s plot experiment (detailed in the Experimental Section). By measuring the fluorescence intensity of mixtures with varying probe-to-metal ion ratios, we observed 1:1 complex formation (Figure S13). Subsequently, we calculated *K*_d_ for each metal ion using fluorescence titration data. The calculated *K*_a_ values were 3.11 × 10^5^ M^–1^ for Al­(III) and 8.32 × 10^6^ M^–1^ for Hg­(II), indicating a relatively strong binding affinity between the probes and the metal ions (Figure S14). These results highlight the potential of these probes as effective sensors for metal ions. The interaction morphology between a sensor and its target has a significant influence on its performance. In this study, the probes were primarily examined as selective ion binders. According to previous reports, two possible binding mechanisms were reported for probes: binding to heteroatoms or ring opening of the rhodamine ring induced by this binding. To elucidate the interaction between probes and mercury and aluminum ions, we employed ^1^H NMR titration (Figure S15). The significant spectral changes observed, including peak shifts and the emergence of new peaks, strongly indicate the formation of a ring-open complex. Namely, the OH peak at about 9.5 ppm, which denotes the enol group produced as a result of the **P1**–Al­(III) interaction, is particularly noticeable (Figure S15). The fact that this similar peak did not occur in the **P2**–Al­(III) interaction strengthens the fact that there is no ring opening here. In general, the interaction of aluminum and mercury ions with the N and O groups in the probes’ rhodamine ring is responsible for these changes. To gain a deeper understanding of the binding morphology, mass spectrometry was conducted alongside NMR analysis to examine the probe–metal complexes. High-resolution mass spectrometry (HRMS, ESI-TOF) results revealed the following: for the **P1** + Al­(III) complex, the [M ]^+^ ion was observed at *m*/*z* 856.4277 (calculated for C_51_H_62_N_4_O_4_AlCl: 856.4269); and for the **P1** + Hg^2+^ complex, the [M ]^2+^ ion appeared at *m*/*z* 513.2813 (calculated for C_51_H_63_N_4_O_4_HgCl: 513.2093), as shown in Figure S16. These results support the formation of the respective metal–probe complexes.

In the initial phase of this study, we investigated probes **P1**/**P2** as potential sensors for detecting aluminum, mercury, cyanide, and glutathione in aqueous environments. To evaluate the practical utility of these sensor candidates, we compared their performances to those of previously reported sensors for the same targets. As summarized in Table 1, the probes exhibit comparable performance to existing sensors, making them promising tools for detecting aluminum, mercury, cyanide, and glutathione in MeOH/H_2_O and water.^6,9,43–57^

**Table 1:** Comparison of Some Al­(III), Hg­(II), CN^–^, and GSH Selective Chemosensors

reference	sensing ions	LOD
[^6^]	Hg(II)	0.41
	Al(III)	1.35
	CN^–^	0.36 μM
[^9^]	GSH	544 nM
[^43^]	Hg(II)	18.0 μM
[^44^]	Hg(II)	14.5 μM
[^45^]	Hg(II)	0.10 μM
[^46^]	Hg(II)	18.0 μM
[^47^]	Al(III)	4.32 μM
[^48^]	Al(III)	10.0 μM
[^49^]	Al(III)	7.41 μM
[^50^]	Al(III)	10.0 μM
[^51^]	CN^–^	2.15 μM
[^52^]	CN^–^	1.20 μM
[^53^]	CN^–^	2.41 μM
[^54^]	CN^–^	4.30 μM
[^55^]	GSH	4.30 μM
[^56^]	GSH	30.0 nM
[^57^]	GSH	37.0 nM
this study	Al(III)	857 nM
	Hg(II)	389 nM
	CN^–^	680 nM
	GSH	1.21 μM

### 2.3 Design of the Molecular Memory Unit

One significant advantage of these organic probes is their ability to reversibly switch their fluorescence on and off in response to specific analytes. Experimental results demonstrate that adding Hg­(II)/Al­(III) ions, followed by GSH and cyanide ions, to probes resulted in repeated fluorescence activation and deactivation cycles at approximately 580 nm (Figures 3 and S17). This reusability allows the probes to be utilized for up to 12 cycles of Hg­(II)/Al­(III) ions and subsequent GSH/CN^–^ detection. The interaction between the probes and metal ions appears to form a stable complex with GSH and CN^–^, as adding excess GSH and CN^–^ ions led to increased fluorescence. This unique property of the probes enabled us to explore their potential for constructing a molecular logic gate. In this logic system, “on” and “off” fluorescence states were assigned the values “1” and “0”, respectively. In the absence of metal ions (Input 1) or GSH/CN^–^ (Input 2), the probes remained “off” (output logic “0”). However, the addition of metal ions (In1) activated the fluorescence (output logic “1”). Conversely, the introduction of GSH or CN^–^ (In2) to **P1**–Hg­(II) and **P1**/**P2**–Al­(III) complexes deactivated the fluorescence (output logic “0”). Additionally, the bistability behavior of probes **P1** and **P2** in their “on–off” states highlights their nonvolatile memory characteristics (Figure 3, inset scheme). Especially remarkable is the long-term maintenance of the “on–off” state, which enables several “write–read–erase–read” cycles. In other words, this indicates that technology allows for continuous writing with little impact on the optical or spectral output quality. Consequently, this kind of sequential molecular logic circuit runs, and conventional logic circuits are based on semiconductors. This opens up exciting possibilities for future advancements in molecular microprocessors, especially for use as memory components in integrated logic systems.

**Figure 3:**
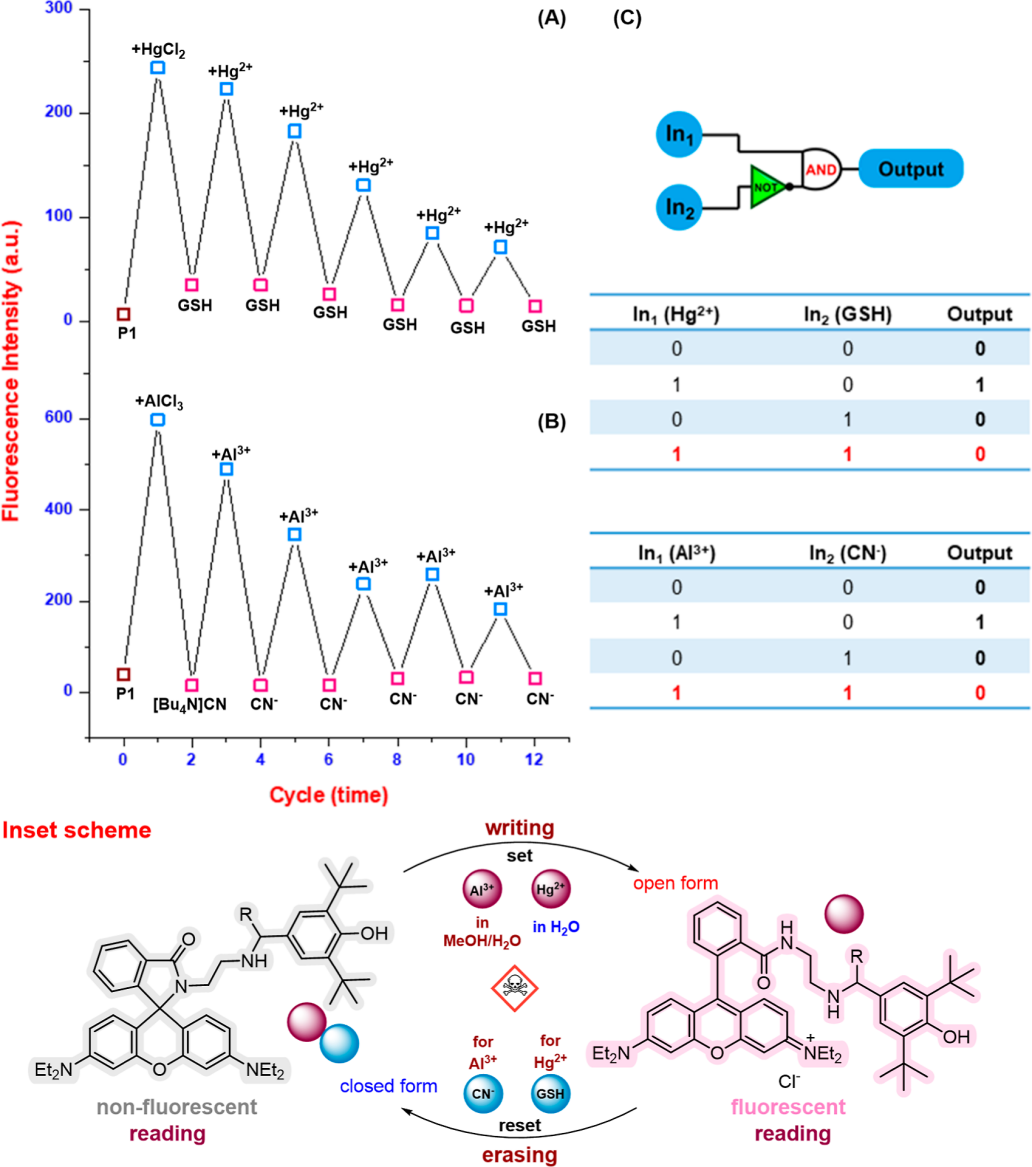
Reversible switching of the fluorescence intensity of **P1**–Hg­(II)/GSH (A) and **P1**–Al­(III)/[Bu_4_N]­CN (B) and (C) the “IMPLICATION” logic gate. Inset scheme: Reversible logic processes with “reading–writing–reading–erasing–reading” activities are revealed by the feedback loop.

### 2.4 Electrical Properties

Spectroscopic analysis indicates an inverse correlation between the HOMO–LUMO gap (*E*_g_) and chemical reactivity, where a larger gap signifies greater stability, and a smaller gap suggests higher reactivity. The experimental absorbance profiles of the synthesized compounds hint at their potential in photovoltaic applications. To further explore the electronic properties of the probes and their interactions with Al­(III) and Hg­(II) ions, we performed density functional theory (DFT) calculations using the B3LYP functional within the Gaussian 09 package. For Al and Hg atoms, the LANL2DZ effective core potential (ECP) and basis set were employed, while the 6–311G­(d,p) basis set was used for all other atoms. Time-dependent DFT (TD-DFT) calculations at the same level provided the corresponding Frontier molecular orbitals (MOs) (Figures 4, S18, S19).^58^ The calculated gas-phase band gap (*E*_g_) values agreed well with the experimental findings. These small organic probes (excluding the closed forms of **P1/P2**) exhibit electronic characteristics suitable for electrical and photophysical devices. The lower *E*_g_ values and UV–vis absorbance of the open forms of probes (**P1/P2**) further support their potential in these applications. Furthermore, the calculated E_g_ and UV–vis values exhibited significant changes upon metal ion interaction: **P1** (4.796 eV/287.59 nm), P1–Al­(III) (2.455 eV/501.23 nm), **P1**–Hg­(II) (2.6544 eV/473.69 nm), **P2** (4.582 eV/312.90 nm), and **P2**–Al­(III) (2.369 eV/571.80 nm) (Figures 4 and S19). The observed decrease in band gaps and the red shift in UV–vis absorption spectra confirm that the interaction with metal ions induced ring opening in the closed-form probes, resulting in a “turn-on” effect.

**Figure 4:**
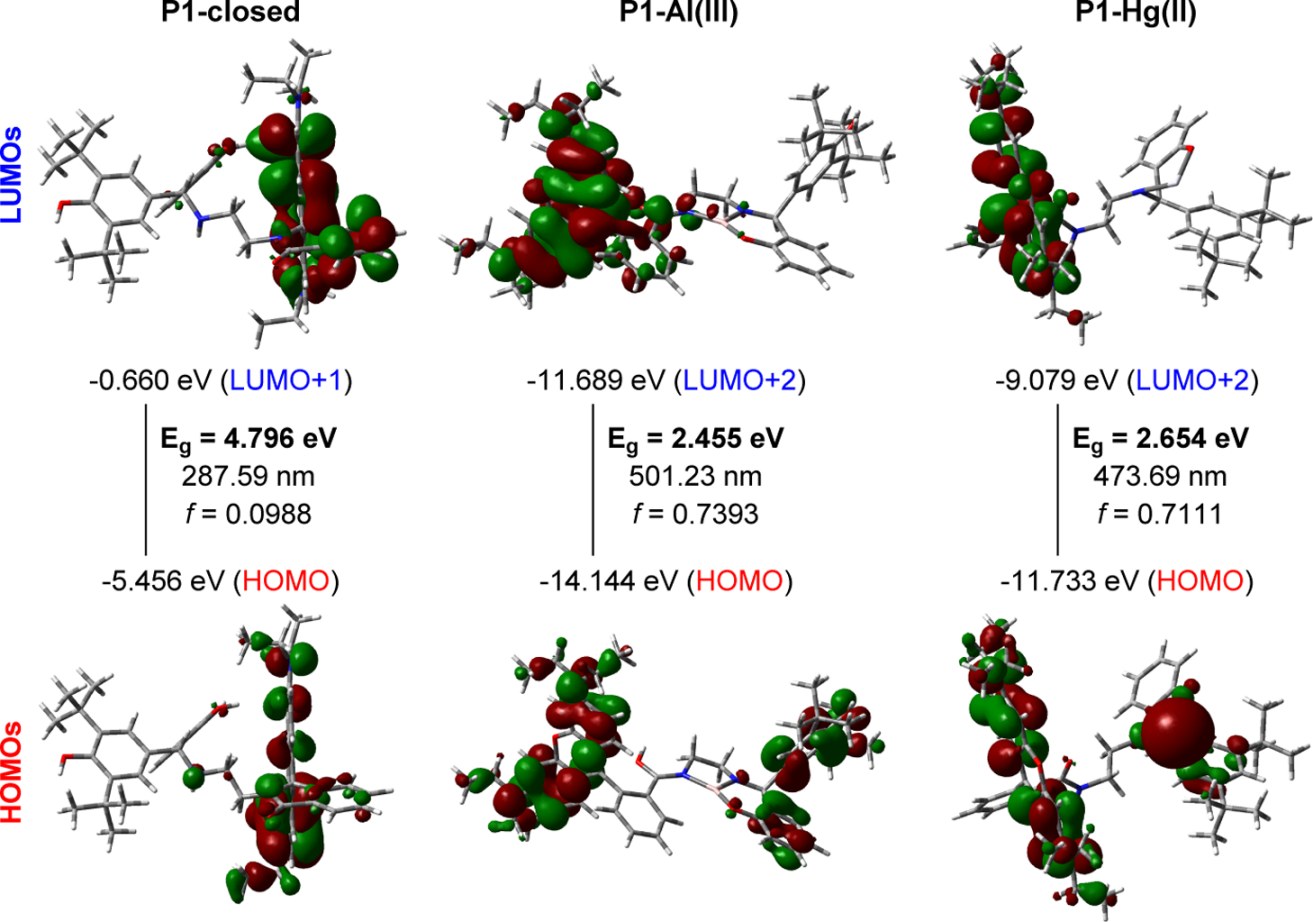
HOMO/LUMO distributions of **P1**-closed/**P1**–Al­(III)/**P1**–Hg­(II).

### 2.5 Practical Application

Simple techniques, such as colorimetric tests and filter paper assays, can effectively complement advanced instrumentation for evaluating potential sensor candidates. We employed these methods in conjunction with real water samples to assess our promising sensor candidates. Initially, we examined the color-changing properties of **P1** in various solvents with different metal ions (Figure 5). **P1** exhibited a distinct color change in the presence of mercury in water and aluminum in MeOH/H_2_O (v/v:1/1), indicating its potential as a visual sensor for these metals in specific environments. To develop a user-friendly test for the detection of Hg­(II)/GSH and Al­(III)/CN^–^, we created test strips by impregnating filter paper with a **P1** solution. These strips displayed a rapid and visible color change when exposed to solutions containing mercury or aluminum ions, particularly under sunlight or UV light. Moreover, we observed that test papers stained with **P1**–Hg­(II) or **P1**–Al­(III) rapidly lost color upon exposure to GSH and cyanide, respectively. These color changes signified the presence of GSH and cyanide, making these dyes particularly valuable for cyanide detection in waste or environmental samples.

**Figure 5:**
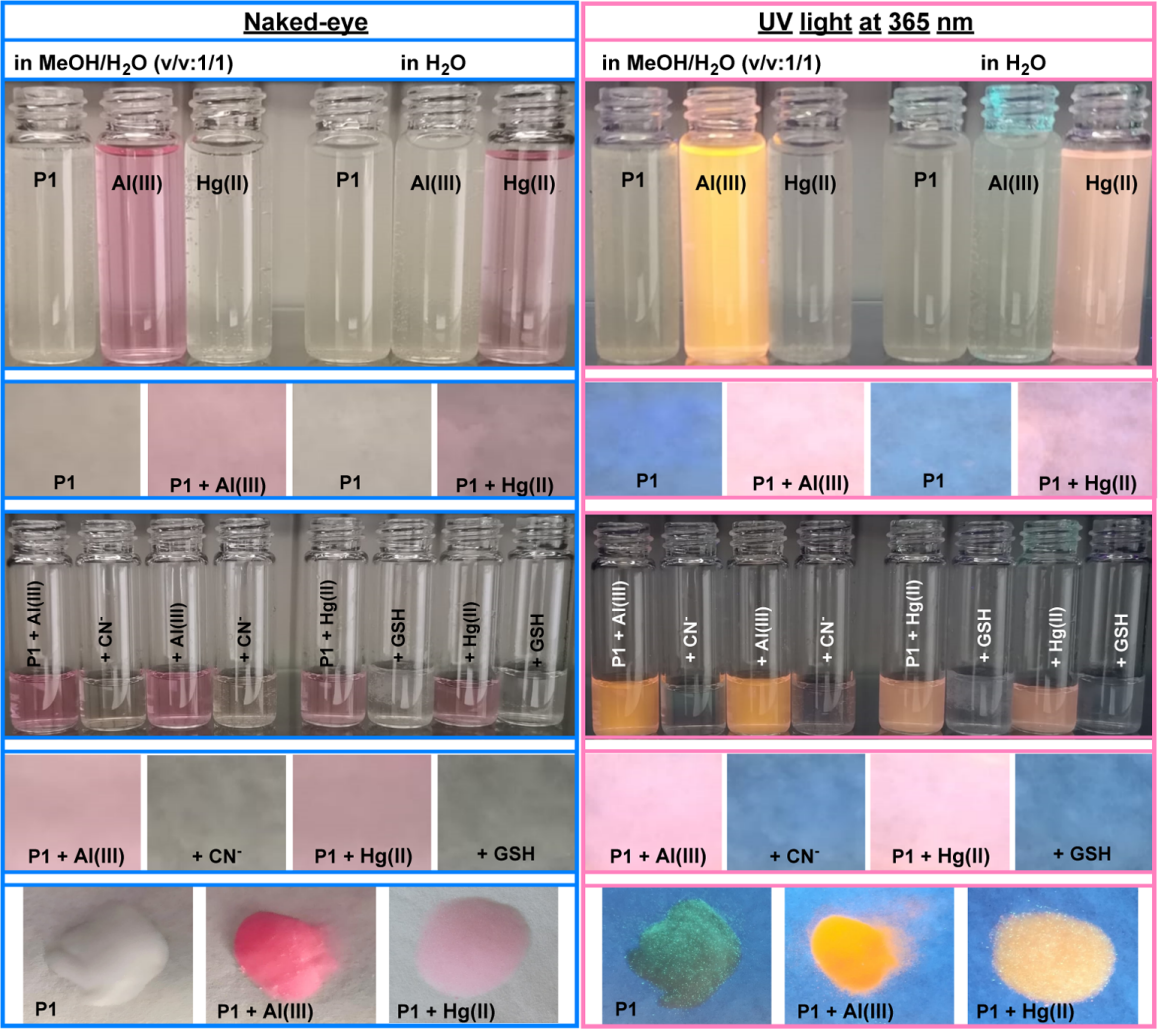
Naked eye and UV light at 365 nm color changes of **P1** in the presence of metal ions in MeOH/H_2_O (v/v: 1/1)/in H_2_O, the photographs depicting the colorimetric response of **P1** systems with filter paper, and silica gel demonstrating a colorimetric and fluorescence response of **P1** to Al­(III) and Hg­(II) ions in a solid medium.

In addition to paper-based detection, probe **P1** also successfully detected Al­(III) and Hg­(II) using a solid-state method with silica gel as a substrate. Silica gel (100–200 mesh) was mixed with a 100 mM MeOH solution of **P1** and dried under reduced pressure, resulting in a colorless solid. This **P1**-coated silica gel rapidly turned dark pink and pale pink upon exposure to 100 mM MeOH/H_2_O (v/v:1/1) solutions of Al­(III) and water solutions of Hg­(II), respectively, further demonstrating strong colorimetric responses of **P1** to aluminum and mercury ions. Both the paper strip and silica gel tests confirmed P1’s specificity and sensitivity, highlighting its potential for real-world applications. These results suggest that **P1**-based test kits could provide a practical and cost-effective way to quickly identify Al­(III) or Hg­(II) ions in various environmental and analytical contexts. The ease of use and visual detection of **P1** make this approach particularly well suited for field detection where sophisticated lab equipment is unavailable. In conclusion, this straightforward and cost-effective method provides a visual approach for detecting mercury, aluminum, GSH, and cyanide ions.

### 2.6 Real-World Applications

To evaluate the practical applicability of the developed probes, recognition studies were conducted as recognition study experiments to assess the probes’ practicality in real-world water samples (tap water, groundwater, and drinking water) (Figure S20). These samples were spiked with varying concentrations of Al­(III) ions and analyzed using fluorescence spectroscopy. As detailed in Table 2, detectable levels of Al­(III) ions were present in all three types of water samples. The recovery rates ranged from 97.3% to 106.0%. The basis for percentage values and the practice of calculating recovery values are detailed in the Experimental Section. These results underscore the probes’ high accuracy and precision in quantifying Al­(III) ions in diverse water matrices.

**Table 2:** Analytical Results for the Determination of AI­(III) in Real-Water Samples

sample	Al(III) added (μmol L^–1^)	Al(III) found (μmol L^–1^)	recovery (%)
tap water	0.0	0.2787	0.0
	0.8	1.0917	101.2
	1.3	1.6229	102.7
	2.0	2.3563	103.4
ground water	0.0	0.1996	0.0
	0.8	0.9972	99.0
	1.3	1.5540	104.0
	2.0	2.3398	106.0
bottled water	0.0	0.1791	0.0
	0.8	0.9534	97.3
	1.3	1.4926	101.0
	2.0	2.9820	104.0

## 3 Conclusion

In conclusion, we have some exciting findings from rhodamine hybrids containing *para*-quinone methide groups. These molecules have the potential for applications in sensors.

### 3.1 Sensing Capabilities

Probes (**P1** and **P2**) can be sensors for specific metal ions like mercury and aluminum in water or MeOH/H_2_O (v/v:1/1). The LOD values were shallow: 389 nM for mercury ions and 857 nM for aluminum ions. Moreover, these probes can also be used to detect other molecules like cyanide and glutathione but in a different way. Probe **P1** initially lights up with metal ions present and then turns off (fluorescence quenching) when cyanide or glutathione is added. The LOD values were again impressive: 680 nM for cyanide and 1.21 μM for glutathione.

### 3.2 Practical Application

The study also explored using these sensors in practical settings. Color changes in filter paper and water samples treated with probes were easily observed, indicating the potential for environmental monitoring of mercury and aluminum.

### 3.3 Real-World Applications

Additionally, experiments in recognition studies to evaluate the feasibility of probe water were conducted in real-world water samples including tap water, groundwater, and drinking water.

## 4 Experimental Section

### 4.1 Synthesis of Probes **P1** and **P2**

To a solution of rhodamine B ethylenediamine (**Rh–NH**_**2**_, 100 mg, 0.2 mmol) in HFIP (2.00 mL), *p*-quinone methides (***p*QMs**, 0.2 mmol) were added, and the mixture was stirred at room temperature for 12 h. After the reaction was complete (as monitored by TLC), the solvent was evaporated under reduced pressure. The crude mixture was purified by thin-layer chromatography (eluted with petroleum ether/ethyl acetate) to give the desired product. **P1**: Prepared according to the general procedure. TLC (PE/EA = 5:1) gave the product as a brown solid (130 mg, 79% yield). ^1^H NMR (400 MHz, CDCl_3_): δ 7.93–7.87 (m, 1H), 7.48–7.42 (m, 2H), 7.12–7.01 (m, 4H), 6.78–6.72 (m, 2H), 6.65 (t, *J* = 7.4 Hz, 1H), 6.46 (d, *J* = 8.9 Hz, 2H), 6.41 (d, *J* = 8.9 Hz, 2H), 6.37–6.33 (m, 2H), 6.27–6.14 (m, 2H), 5.14 (s, 1H), 4.69 (s, 1H), 3.37–3.24 (m, 10H), 2.43 (t, *J* = 5.9 Hz, 2H), 1.38 (s, 18H), 1.15 (q, *J* = 7.1 Hz, 12H). ^13^C NMR (101 MHz, CDCl_3_): δ 169.0, 158.2, 153.8, 153.6, 153.5, 153.3, 149.0, 149.0, 136.0, 132.8, 131.2, 129.3, 128.9, 128.7, 128.4, 128.3, 125.4, 124.5, 124.0, 123.1, 118.9, 116.9, 108.5, 108.3, 105.6, 105.5, 98.0, 97.92, 67.34, 65.3, 46.9, 44.6, 39.7, 34.6, 30.5, 12.9 (Figure S2). HRMS (ESI-TOF) *m*/*z*: [M + H]^+^ calcd for C_51_H_63_N_4_O_4_, 795.4844; found, 795.4871 (Figure S4). **P2**: Prepared according to the general procedure. TLC (PE/EA = 5:1) gave the product as a pink solid (60 mg, 31% yield). ^1^H NMR (400 MHz, CDCl_3_): δ 7.88 (dd, *J* = 5.6, 2.7 Hz, 1H), 7.62 (d, *J* = 2.3 Hz, 1H), 7.45–7.29 (m, 4H), 7.24–6.90 (m, 14H), 6.42 (dd, *J* = 8.8, 3.0 Hz, 2H), 6.36–6.32 (m, 2H), 6.22 (dt, *J* = 8.8, 3.0 Hz, 2H), 5.05 (s, 1H), 4.49 (s, 1H), 3.29 (q, *J* = 7.2 Hz, 10H), 2.35 (s, 2H) 1.38 (s, 18H), 1.16–1.10 (m, 12H). ^13^C NMR (100 MHz, CDCl_3_): δ 186.6, 154.0, 153.5, 153.4, 149.3, 148.9, 148.2, 147.1, 146.9, 143.1, 135.9, 135.5, 132.5, 132.2, 129.8, 129.3, 129.0, 128.6, 128.1, 128.0, 125.9, 124.5, 124.4, 124.3, 124.1, 124.0, 123.0, 122.5, 121.4, 108.3, 98.0, 97.9, 66.4, 46.6, 44.6, 40.6, 35.7, 35.2, 34.6, 30.6, 29.8, 29.77, 12.9 (2C signal overlaps) (Figure S3). HRMS (ESI-TOF) *m*/*z*: [M]^+^ calcd for C_63_H_71_N_5_O_3_, 945.5551; found, 945.5534 (Figure S4).

### 4.2 Procedures of Recognition Measurement

#### 4.2.1 UV–vis and Fluorescence Studies of Probes with Various Ions

To investigate the interaction with ions, a 10 μM aqueous solution of probes was prepared in a quartz cell. Baseline UV–vis absorbance and fluorescence spectra were acquired. All experiments were then performed by the stepwise addition of a 1 × 10^–2^ M aqueous solution of the selected ions (1 equiv per addition). After each addition, the changes in UV–vis absorbance intensity were recorded at room temperature within a 2 min time frame.

#### 4.2.2 UV–vis and Fluorescence Titration Probes with HgCl_2_/AlCl_3_/[Bu_4_N]­CN/GSH

Stock solutions of the probes (1 × 10^–2^ M in MeOH) and the analytes HgCl_2_, AlCl_3_, [Bu_4_N]­CN, and GSH (1 × 10^–2^ M in H_2_O) were prepared. Spectroscopic titrations were conducted using a 10 μM solution of probes in either MeOH/H_2_O (v/v: 1/1) for Al­(III) and CN^–^ detection or H_2_O for Hg­(II) and GSH detection. UV–vis and fluorescence spectra were recorded after each addition of the respective analyte solution. All titrations were repeated at least twice to confirm the consistency of the data.

#### 4.2.3 Job’s Plot Measurement

Stock solutions (1 × 10^–2^ M) of the rhodamine–quinone methide probes were prepared in MeOH/H_2_O (v/v: 1/1) for Al­(III) and in H_2_O for Hg­(II). A dilution series of the probe was created by taking volumes ranging from 0.0 to 5.00 mL of the 1 × 10^–2^ M probe solution (in 0.50 mL increments) and diluting to 5.00 mL with the appropriate solvent. Similarly, a 1 × 10^–2^ M stock solution of HgCl_2_ and AlCl_3_ was prepared in H_2_O, and volumes ranging from 0.0 to 5.00 mL (in 0.50 mL increments, inversely related to the probe volumes) were added to the probe solutions to maintain a total volume of 5.00 mL. After mixing, absorbance spectra were recorded at room temperature.

#### 4.2.4 Determination of the Detection Limit

Fluorescence measurements were recorded for all solutions containing HgCl_2_, AlCl_3_, [Bu_4_N]­CN, or GSH. The detection limit for each analyte was calculated from the fluorescence titration data using the 3σ/k equation. In this equation, “σ” is the standard deviation of the blank measurements, and “*k*” is the slope of the linear fit of the fluorescence response to the analyte concentration.

#### 4.2.5 Determination of the Association Constant

The association constant (*K*_d_) was determined using the Benesi–Hildebrand equation applied to fluorescence titration data according to the following relationship:

$$\frac{1}{F-F_{0}}=\frac{1}{\left\{K_{d}\left(F_{\max}-F_{0}\right){\left[M^{x+}\right]}^{n}\right\}}+\frac{1}{F_{\max}-F_{0}}$$

In this equation, *F*_0_ and *F* represent the fluorescence intensities of the receptor without and with the added metal ion [M^*x*+^], respectively, while *F*_max_ is the saturation fluorescence. K_d_ is the association constant, and “*n*” denotes the binding stoichiometry. The value of *K*_d_ was derived from the slope of the linear plot of 1/(*F* – *F*_0_) against 1/[M]^1^.

#### 4.2.6 The pH Measurement

The effect of pH (2–12) on the fluorescence of Probes (10 μM in EtOH) was evaluated in the absence and presence of HgCl_2_ or AlCl_3_ (30 μM in H_2_O) to explore their practical use. Ten samples of the probe solutions were prepared, and where applicable, metal ions were added. The pH of each sample was adjusted between 2 and 12 using HCl or NaOH, and the resulting pH was confirmed with a pH meter and/or pH strips.

#### 4.2.7 The Reversible Switching Studies

Stock solutions of the probes (**P1** or **P2**), metal ions (Al­(III), Hg­(II)), and anions (CN^–^, GSH) were prepared at known concentrations (typically 1 mM) in H_2_O and MeOH. To evaluate the reversibility of the probe response, cycle studies were conducted by alternately introducing metal ions and corresponding counteranalytes (anions) into the probe solution, followed by monitoring the changes in fluorescence. Initially, a 10 μM solution of the probe was prepared in MeOH/H_2_O (v/v.1/1) or H_2_O, and its baseline spectrum was recorded. A stoichiometric or slightly excess amount of metal ion (1.0–1.5 equiv) was then added, and the resulting color or fluorescence change was recorded after 2 min of incubation. Subsequently, an equivalent or excess amount of a competing anion (CN^–^ for Al­(III) or GSH for Hg­(II)) was added to the metal–probe complex, and spectral changes were recorded again after 1–2 min to observe signal reversal. This cycle of metal addition and anion-triggered recovery was repeated for 10 cycles to assess the reversibility and stability of the sensing process.

#### 4.2.8 Preparation of the Filter Paper

Colorimetric cards were developed by using filter paper. A piece of filter paper was incubated at room temperature for 5 h with probes. After incubation, the papers were washed with water and dried at 50 °C. The prepared colorimetric cards were exposed to selected metal ion samples either by immersion or by applying sample droplets directly onto the paper surface. Color changes were then observed and recorded. All experiments were performed in triplicate to ensure reproducibility.

#### 4.2.9 Real-Sample Tests

To evaluate the practical applicability of probe **P1**, real-world water samples, including tap water, groundwater, and drinking water, were analyzed. A stock solution of aluminum ions was first prepared. Aliquots corresponding to final concentrations of 0.0, 0.8, 1.3, and 2.0 μmol L^–1^ were subsequently drawn from this stock and introduced into separate 5 mL solutions containing probe **P1**. Each mixture was incubated at room temperature, and fluorescence measurements were recorded to assess the probe’s response in these real-sample matrices. Specifically, the percentage recovery was determined by comparing the amount detected to the amount originally added using the formula:

Recovery(%)=(Measuredconcentration/Spikedconcentration)×100

## Supplementary Material



## Notes

General experimental methods and characterizations of the materials (PDF)
