# Supplementary material for: Water-Driven Cascade Specific Detection of Al(III)/Hg(II) and CN–/GSH in Real Water Samples
Source: ACS Omega. 2025 Jul 14;10(28):31022–32. doi: 10.1021/acsomega.5c04268 (PMC12290952; doi:10.1021/acsomega.5c04268)
Supplement: Supplementary file 1 [file ao5c04268_si_001.pdf]

Supplementary Data for

Water-Driven Cascade Specific Detection of Al(III)/Hg(II) and CN^-/GSH in Real Water Samples

Rabia Ardahanlı[a,¥], Abdullah S. Hussein[b,c,¥], Ferruh Lafzi[a], Sinan Bayindir[b]*, and Haydar Kilic[a]*

[a]Department of Chemistry, Faculty of Sciences, Ataturk University, 25240, Erzurum, Türkiye
[b]Department of Chemistry, Faculty of Sciences and Arts, Bingol University, 12000, Bingol, Türkiye
[c]College of Education Chemistry Department, Salahaddin University-Erbil, 44001, Erbil, Iraq

***Corresponding author:**

Sinan Bayindir (sbayindir@bingol.edu.tr);

Haydar Kilic (hydk@atauni.edu.tr)

General methods

All chemicals, reagents, and solvents were commercially available from Sigma-Aldrich or Merck. ^{1}H NMR and ^{13}C NMR spectra were recorded on a 400 (100)-MHz Bruker spectrometer and are reported in terms of chemical shift (δ, ppm) with $SiMe_4$ as an internal standard. Data for ^{1}H NMR are recorded as follows: chemical shift (δ, ppm), multiplicity (s: singlet, d: doublet, t: triplet, q: quartet, p: pentet, m: multiplet, bs: broad singlet, bd: broad doublet, qd: quasi doublet) and coupling constant (s) in Hz, integration. Elemental analyses were carried out on a LECO CHNS-932 instrument. Column chromatography was performed on silica gel 60 (230–400 mesh ASTM). The reaction progress was monitored by thin-layer chromatography (TLC) (0.25-mm-thick precoated silica plates: Merck Fertigplatten Kieselgel (60 F254)). HRMS were acquired using a QTOF (Quadrupole time-of-flight) spectrometry instrument. UV-Vis absorption and fluorescence spectra of samples were recorded on a Shimadzu UV-3101PL UV-Vis-NIR spectrometer and Perkin–Elmer (Model LS 55) Fluorescence Spectrophotometer, respectively.

Synthesis of probes P1 and P2

Synthesis of Rh-NH$_2$: Ethylenediamine (1.0 mL, 15.08 mmol) was added dropwise to a solution of rhodamine B (1.0 g, 2.01 mmol) in 25 mL of ethanol. The solution was heated to reflux for 16 h and evaporated to dryness. The resulting residue was dissolved in water and extracted with DCM (2x 15 mL). The combined organic phases were washed with water and dried over Na_2SO_4. The solvent was removed by evaporation, and the remaining solid was dried in vacuo, affording 2-(2-aminoethyl)-3',6'-bis(diethylamino)spiro[isoindoline-1,9'-xanthen]-3-one (**Rh-NH$_2$**) as a pinkish powder (0.96 g, 99%). ^{1}H NMR (400 MHz, $CDCl_3$) δ 7.89 (dd, *J* = 5.6, 3.1 Hz, 1H), 7.43 (dd, *J* = 5.6, 3.1 Hz, 2H), 7.08 (dd, *J* = 5.9, 2.7 Hz, 1H), 6.42 (d, *J* = 8.9 Hz, 2H), 6.36 (d, *J* = 2.6 Hz, 2H), 6.26 (dd, *J* = 8.9, 2.7 Hz, 2H), 3.32 (q, *J* = 7.3 Hz, 8H), 3.18 (t, *J* = 6.7 Hz, 2H), 2.40 (t, *J* = 6.6 Hz, 2H), 1.15 (t, *J* = 7.0 Hz, 12H). ^{13}C NMR (100 MHz, $CDCl_3$) δ 168.9, 153.7, 153.5, 149.0, 132.6, 131.4, 128.9, 128.3, 124.0, 123.0, 108.3, 105.8, 97.9, 65.1, 44.6, 44.1, 41.1, 12.8 (Figure S1). [1.2]

General procedure for the synthesis of P1 and P2: To a solution of rhodamine B ethylenediamine (**Rh-NH$_2$**, 100 mg, 0.2 mmol) in HFIP (2 mL), *p*-Quinone methides (**pQMs**, 0.2 mmol) was added, and the mixture was stirred at room temperature for 12h. After the reaction was complete (monitored by TLC), the solvent evaporation was under reduced pressure. The crude mixture was purified by thin-layer chromatography (eluted with petroleum ether/ethyl acetate) to give the desired product.

Probe P1: Prepared according to the general procedure. TLC (PE/EA=5:1) gave the product as a brown solid (130 mg, 79% yield). ^{1}H NMR (400 MHz, $CDCl_3$) δ 7.93 – 7.87 (m, 1H), 7.48 – 7.42 (m, 2H), 7.12 – 7.01 (m, 4H), 6.78 – 6.72 (m, 2H), 6.65 (t, *J* = 7.4 Hz, 1H), 6.46 (d, *J* = 8.9 Hz, 2H),

6.41 (d, J = 8.9 Hz, 2H), 6.37 – 6.33 (m, 2H), 6.27 – 6.14 (m, 2H), 5.14 (s, 1H), 4.69 (s, 1H), 3.37 – 3.24 (m, 10H), 2.43 (t, J = 5.9 Hz, 2H), 1.38 (s, 18H), 1.15 (q, J = 7.1 Hz, 12H). ^{13}C NMR (101 MHz, $CDCl_3$) δ 169.0, 158.2, 153.8, 153.6, 153.5, 153.3, 149.0, 149.0, 136.0, 132.8, 131.2, 129.3, 128.9, 128.7, 128.4, 128.3, 125.4, 124.5, 124.0, 123.1, 118.9, 116.9, 108.5, 108.3, 105.6, 105.5, 98.0, 97.92, 67.34, 65.3, 46.9, 44.6, 39.7, 34.6, 30.5, 12.9 (Figure S2); HRMS (ESI-TOF) m/z: $[M + H]^+$ calcd. for $C_{51}H_{63}N_4O_4$, 795.4844; found 795.4871 (Figure S4).

Probe P2: Prepared according to the general procedure. TLC (PE/EA=5:1) gave the product as a pink solid (60 mg, 31% yield). ^{1}H NMR (400 MHz, $CDCl_3$) δ 7.88 (dd, J = 5.6, 2.7 Hz, 1H), 7.62 (d, J = 2.3 Hz, 1H), 7.45 – 7.29 (m, 4H), 7.24 – 6.90 (m, 14H), 6.42 (dd, J = 8.8, 3.0 Hz, 2H), 6.36 – 6.32 (m, 2H), 6.22 (dt, J = 8.8, 3.0 Hz, 2H), 5.05 (s, 1H), 4.49 (s, 1H), 3.29 (q, J = 7.2 Hz, 10H), 2.35 (s, 2H) 1.38 (s, 18H), 1.16 – 1.10 (m, 12H). ^{13}C NMR (100 MHz, $CDCl_3$) δ 186.6, 154.0, 153.5, 153.4, 149.3, 148.9, 148.2, 147.1, 146.9, 143.1, 135.9, 135.5, 132.5, 132.2, 129.8, 129.3, 129.0, 128.6, 128.1, 128.0, 125.9, 124.5, 124.4, 124.3, 124.1, 124.0, 123.0, 122.5, 121.4, 108.3, 98.0, 97.9, 66.4, 46.6, 44.6, 40.6, 35.7, 35.2, 34.6, 30.6, 29.8, 29.77, 12.9 (2C signal overlaps) (Figure S3). HRMS (ESI-TOF) m/z: $[M]^+$ calcd for $C_{63}H_{71}N_5O_3$, 945.5551; found 945.5534 (Figure S4).

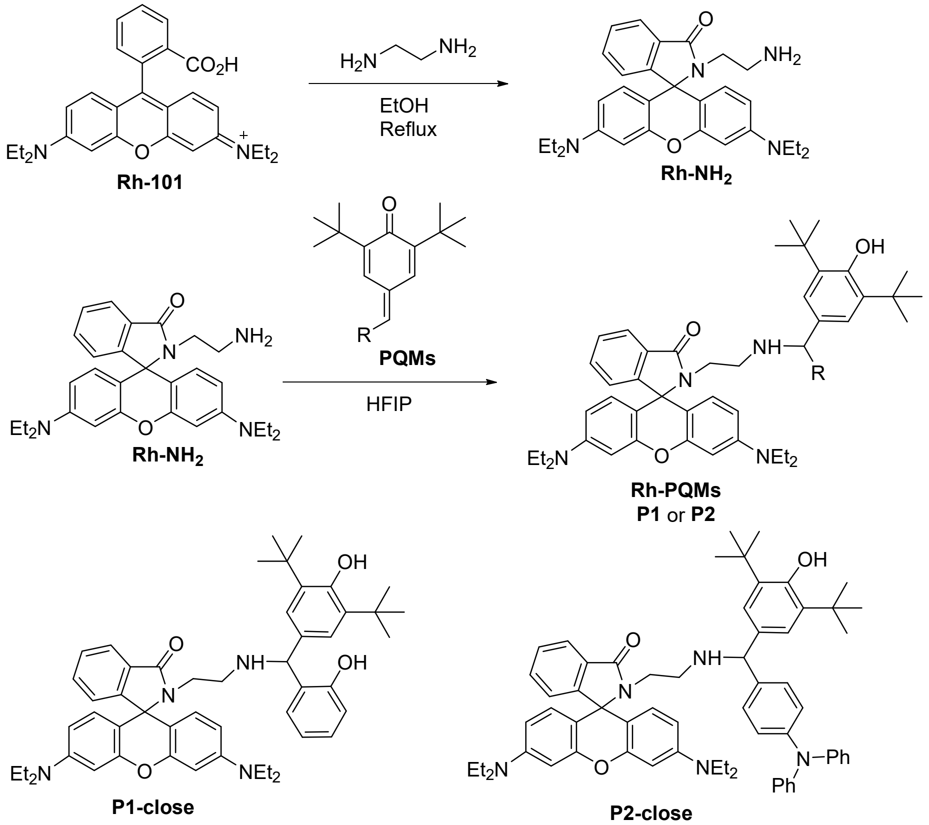

Scheme S1. Synthesis strategies of **P1** and **P2**

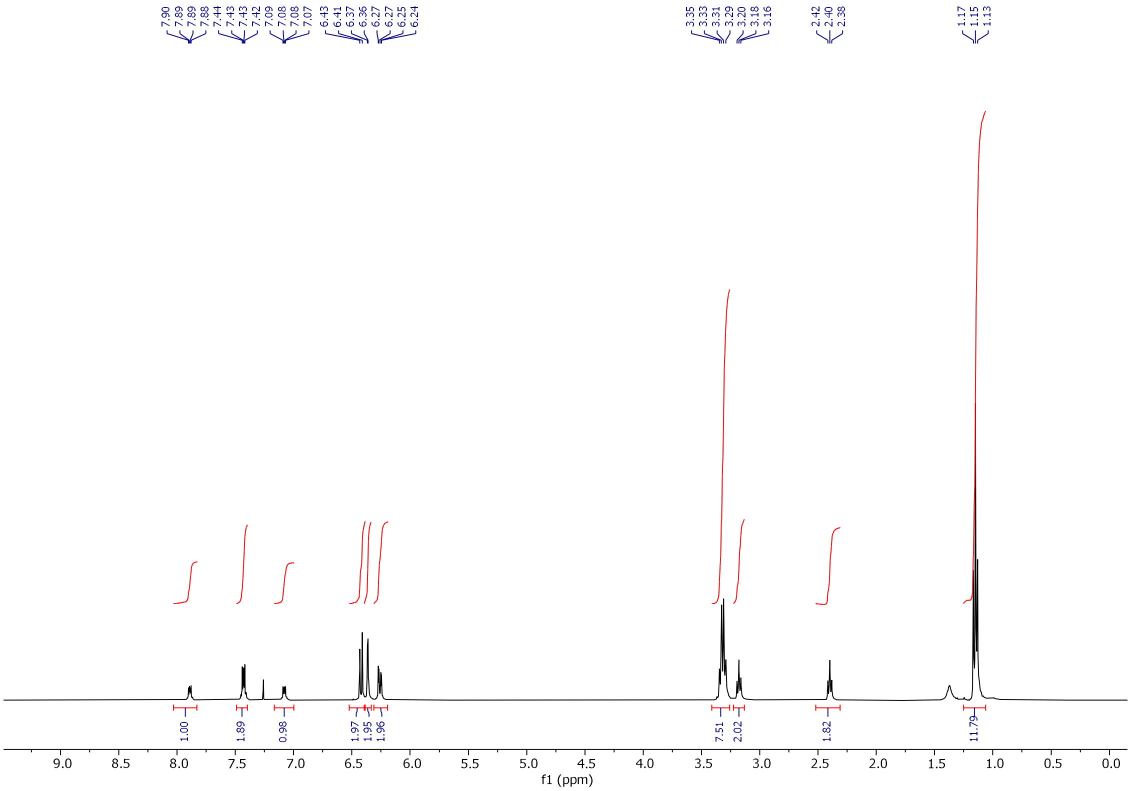

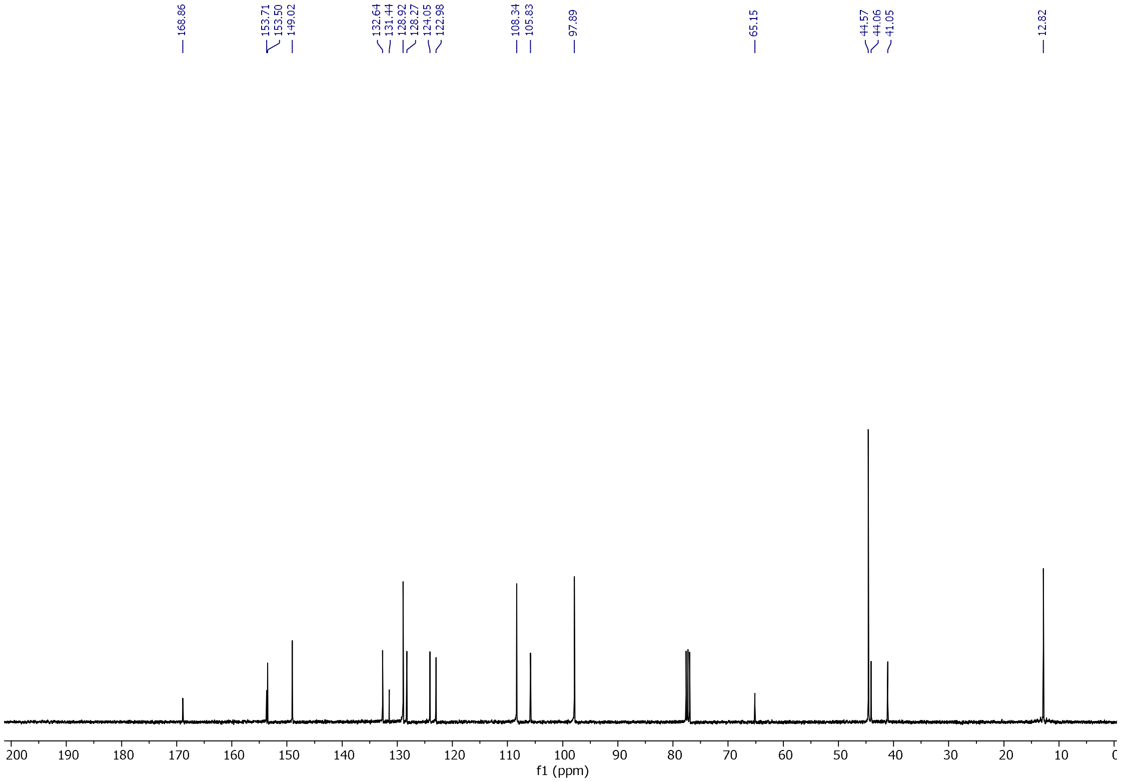

Figure S1. The NMR spectrums of 2-(2-aminoethyl)-3',6'-bis(diethylamino)spiro[isoindoline-1,9'-xanthen]-3-one (**Rh-NH$_2$**) in $CDCl_3$.

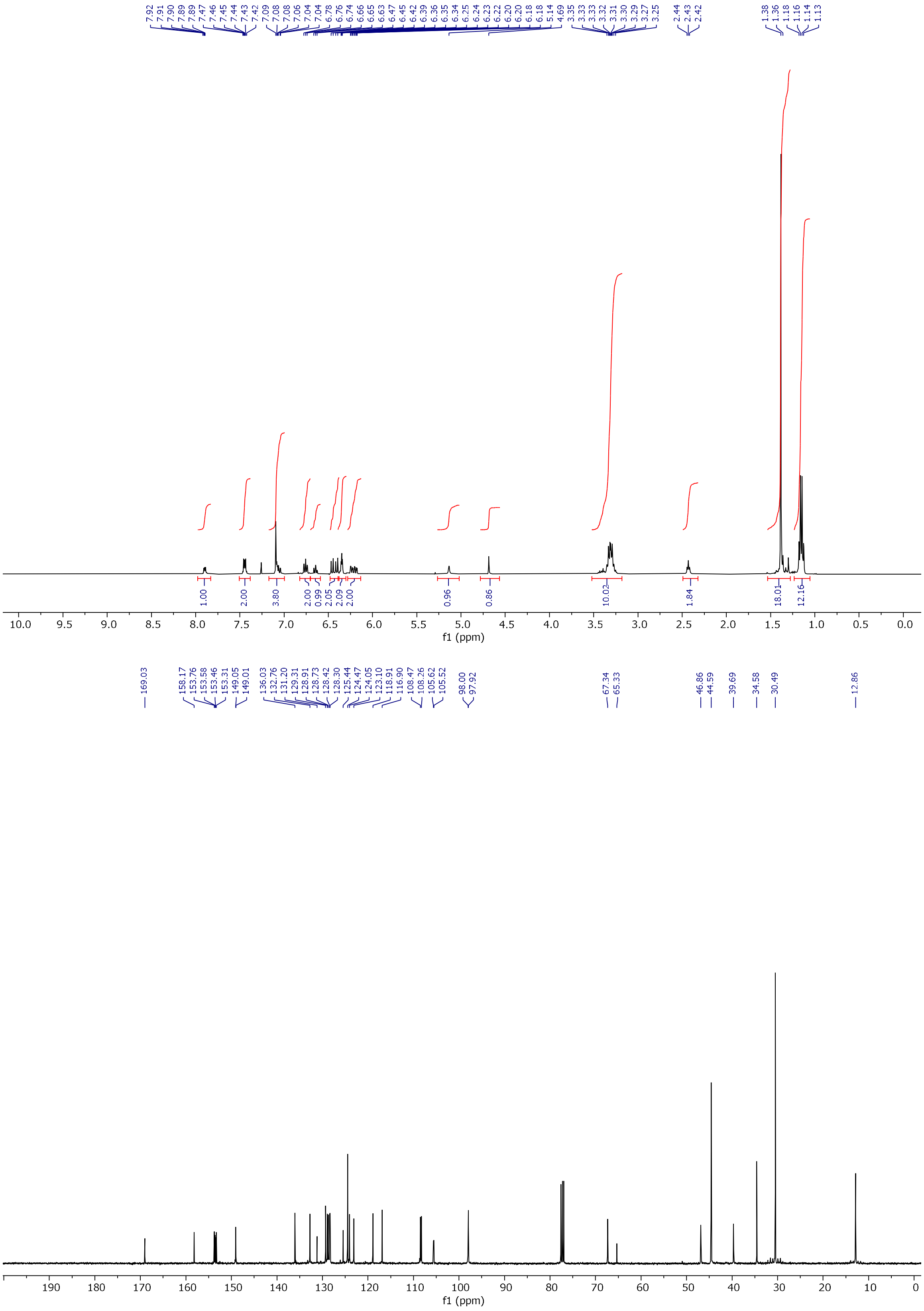

Figure S2. The NMR spectrums of **P1** in $CDCl_3$

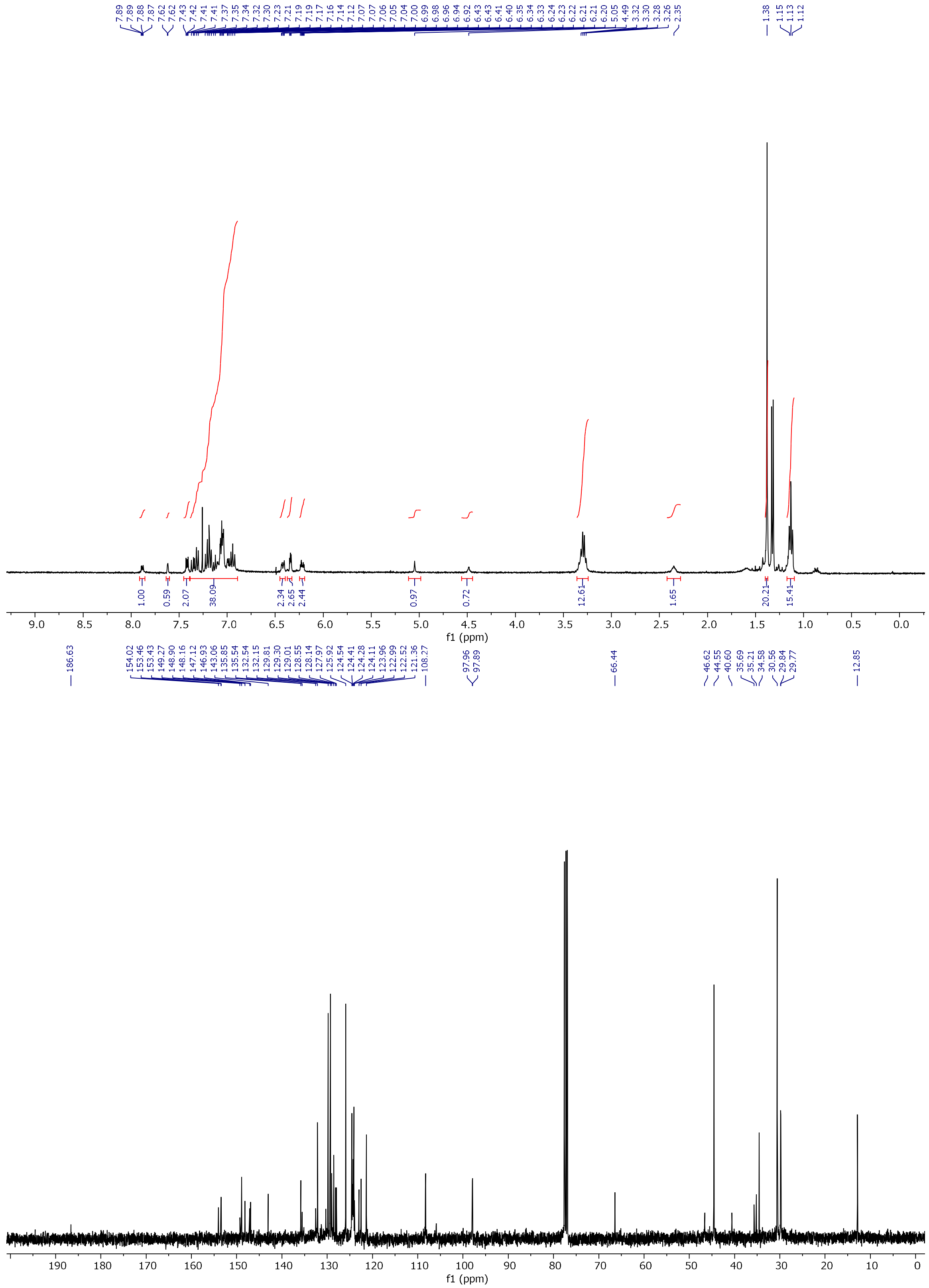

Figure S3. The NMR spectrums of **P2** in $CDCl_3$

P1: HRMS (ESI-TOF) m/z: $[M + H]^+$ calcd for $C_{51}H_{63}N_4O_4$, 795.4844; found 795.4871.

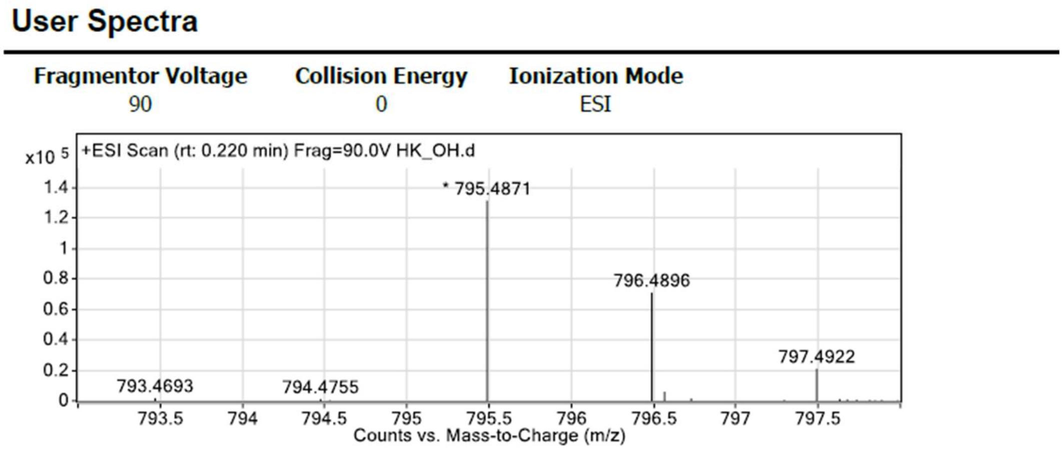

P2: HRMS (ESI-TOF) m/z: $[M]^+$ calcd for $C_{63}H_{71}N_5O_3$, 945.5551; found 945.5534.

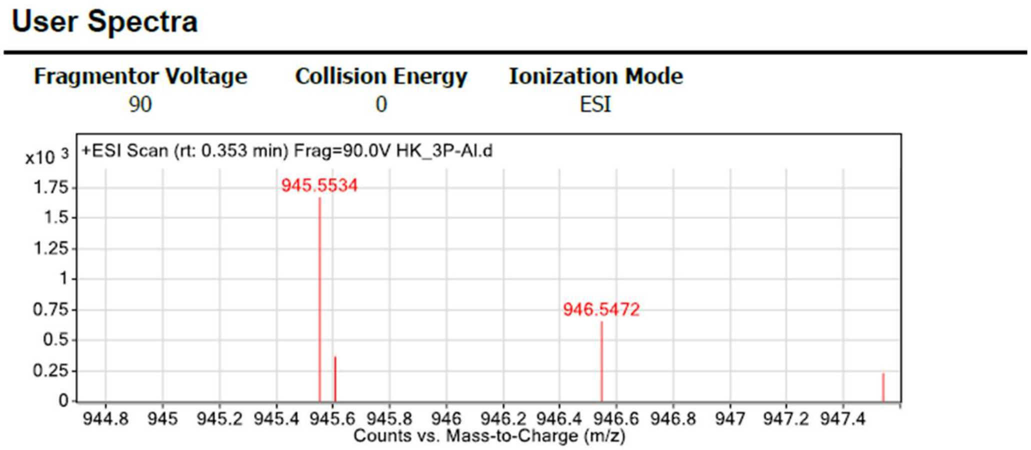

Figure S4. HRMS (ESI-TOF) spectras of P1 (m/z = 1) , and P2 (m/z = 1)

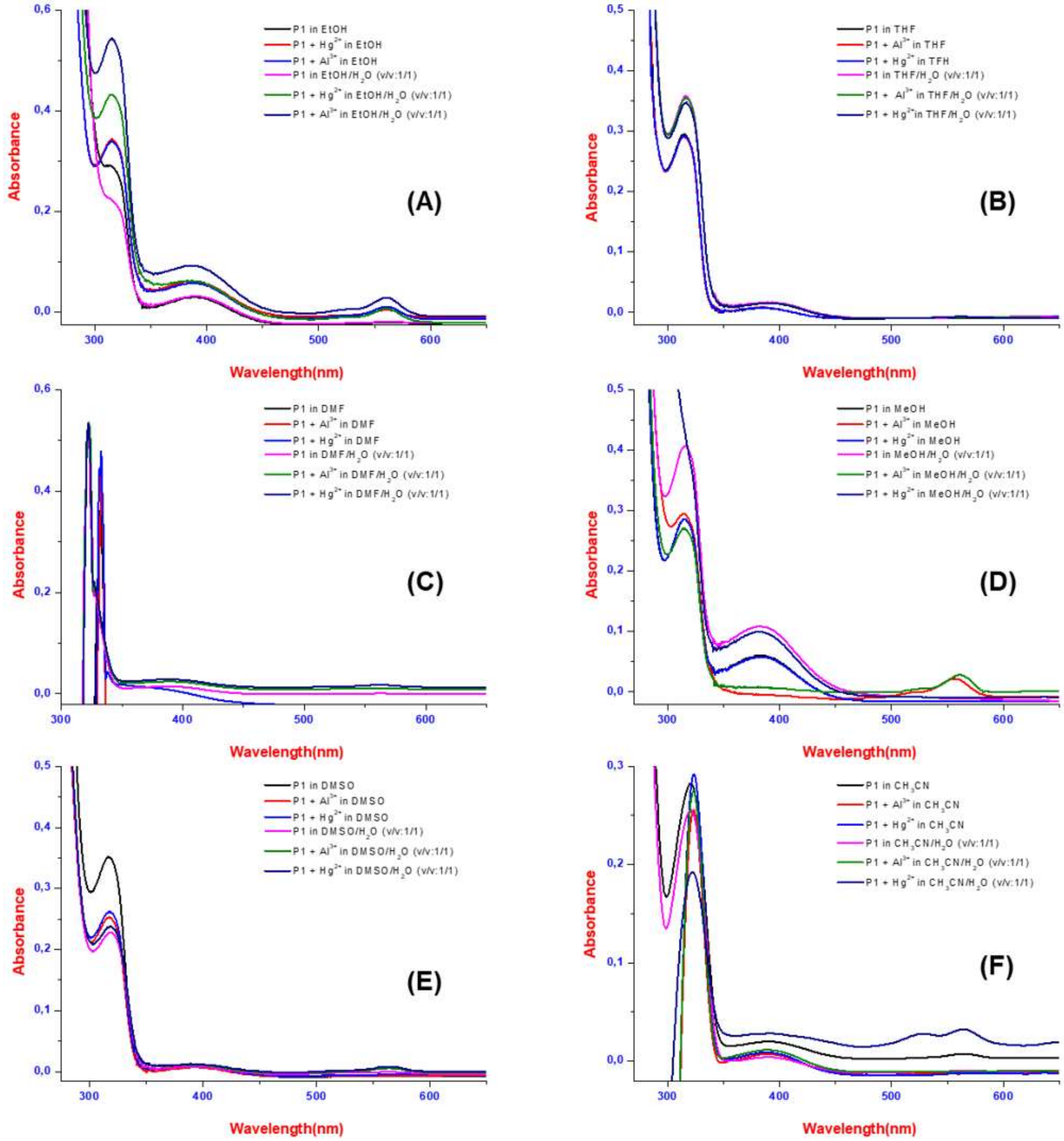

Figure S5. The absorbance spectras of **P1**, **P1**-Al^{3+}, and **P1**-Hg^{2+} in organic solvent (**A**: EtOH, **B**: THF, **C**: DMF, **D**: MeOH, **E**: DMSO, **F**: CH_3CN) / H_2O (v/v: 10/0 and 1/1)

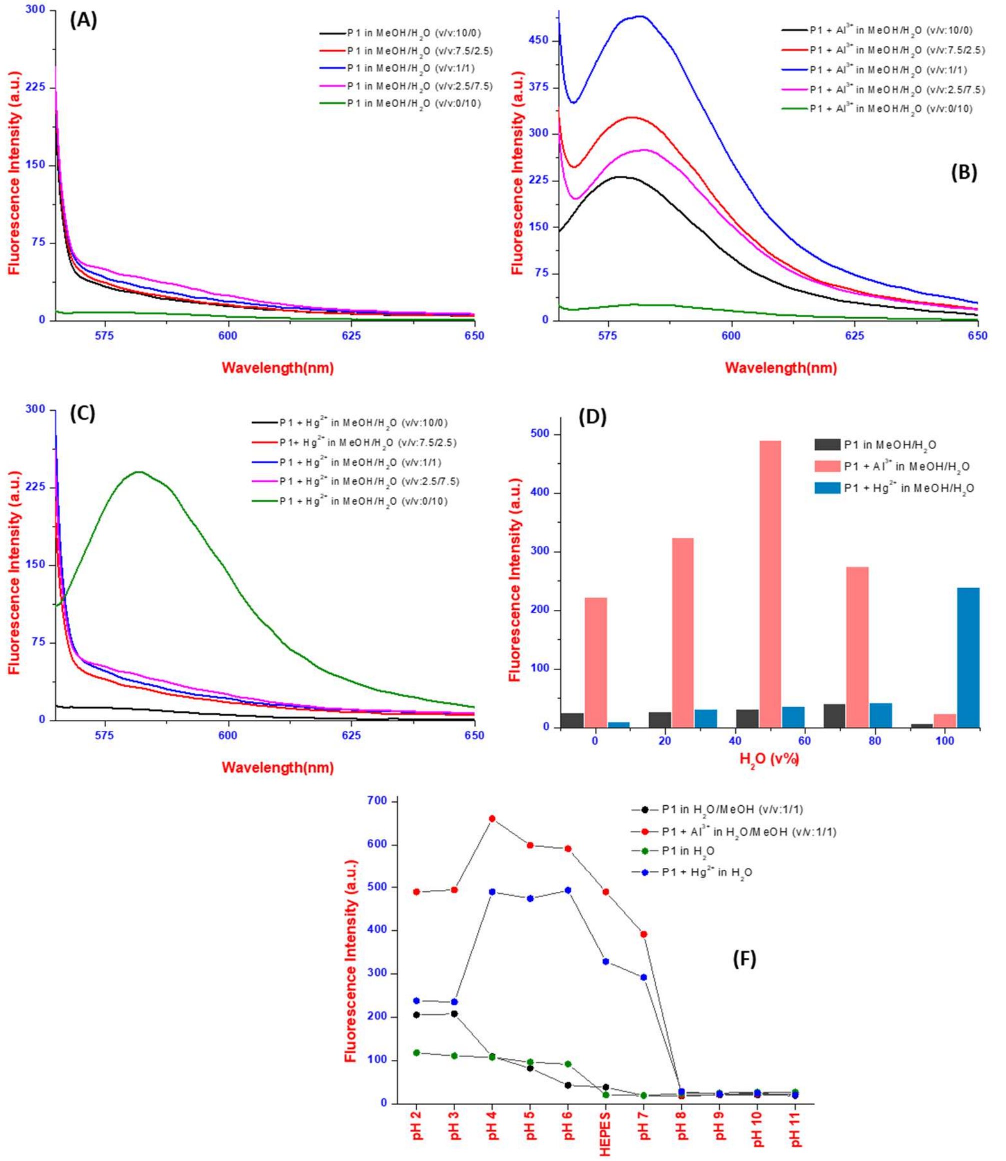

Figure S6. The fluorescence spectras of **P1** (**A**), **P1**-Al^{3+} (**B**), and **P1**-Hg^{2+} (**C**) in MeOH/H_2O (v/v: from 10/0 to 0/10), (**D**) comparative fluorescence Intensities of **P1**, **P1**-Al^{3+}, and **P1**-Hg^{2+} in different water ratios, and (**F**) the fluorescence spectras of probes and probes with [$AlCl_3$] / [$HgCl_2$] at different pH (2–11) in selected solvent systems

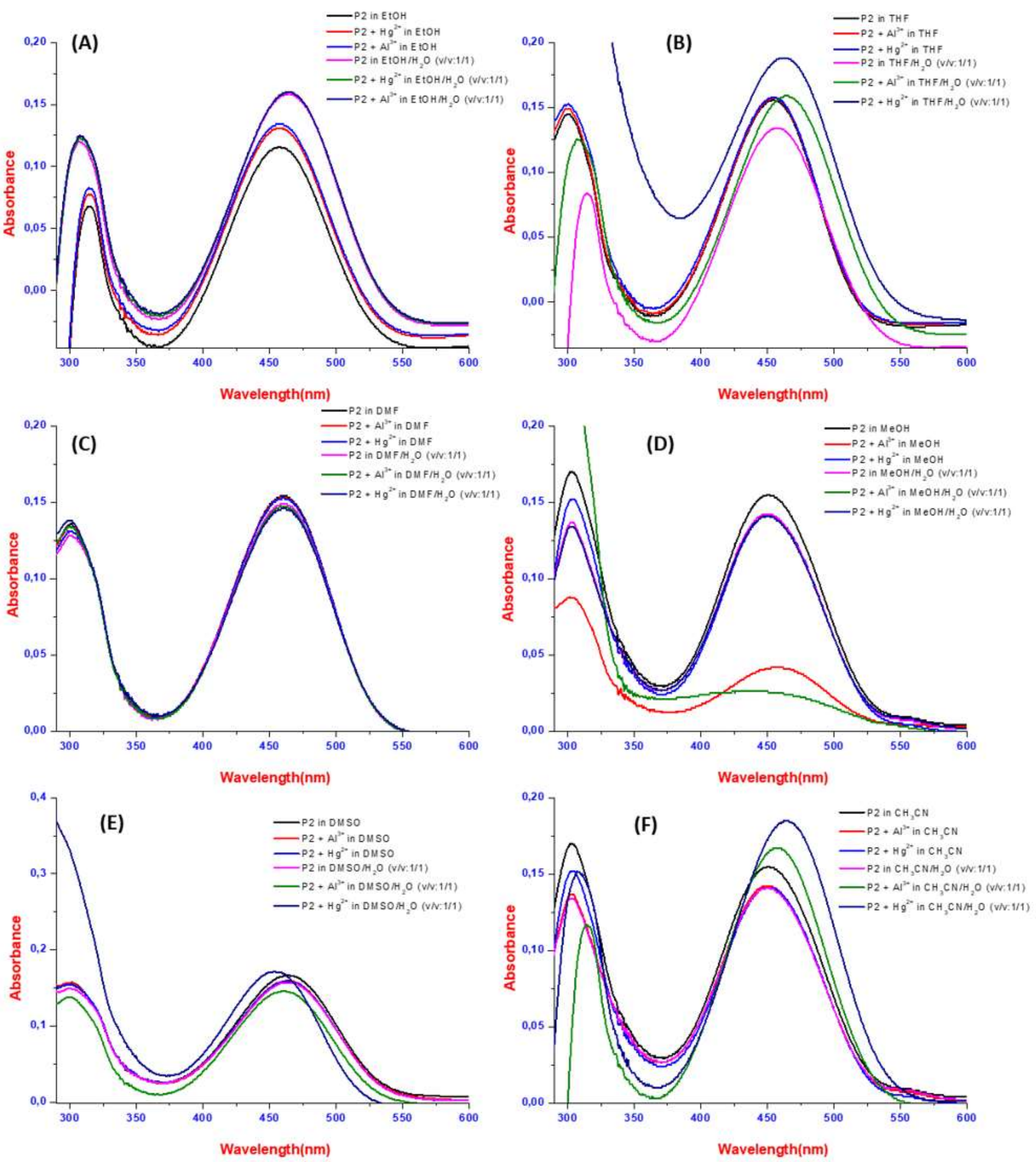

Figure S7. The absorbance spectras of **P2**, **P2**-Al^{3+}, and **P2**-Hg^{2+} in organic solvent (**A**: EtOH, **B**: THF, **C**: DMF, **D**: MeOH, **E**: DMSO, **F**: CH_3CN)/H_2O (v/v: 10/0 and 1/1)

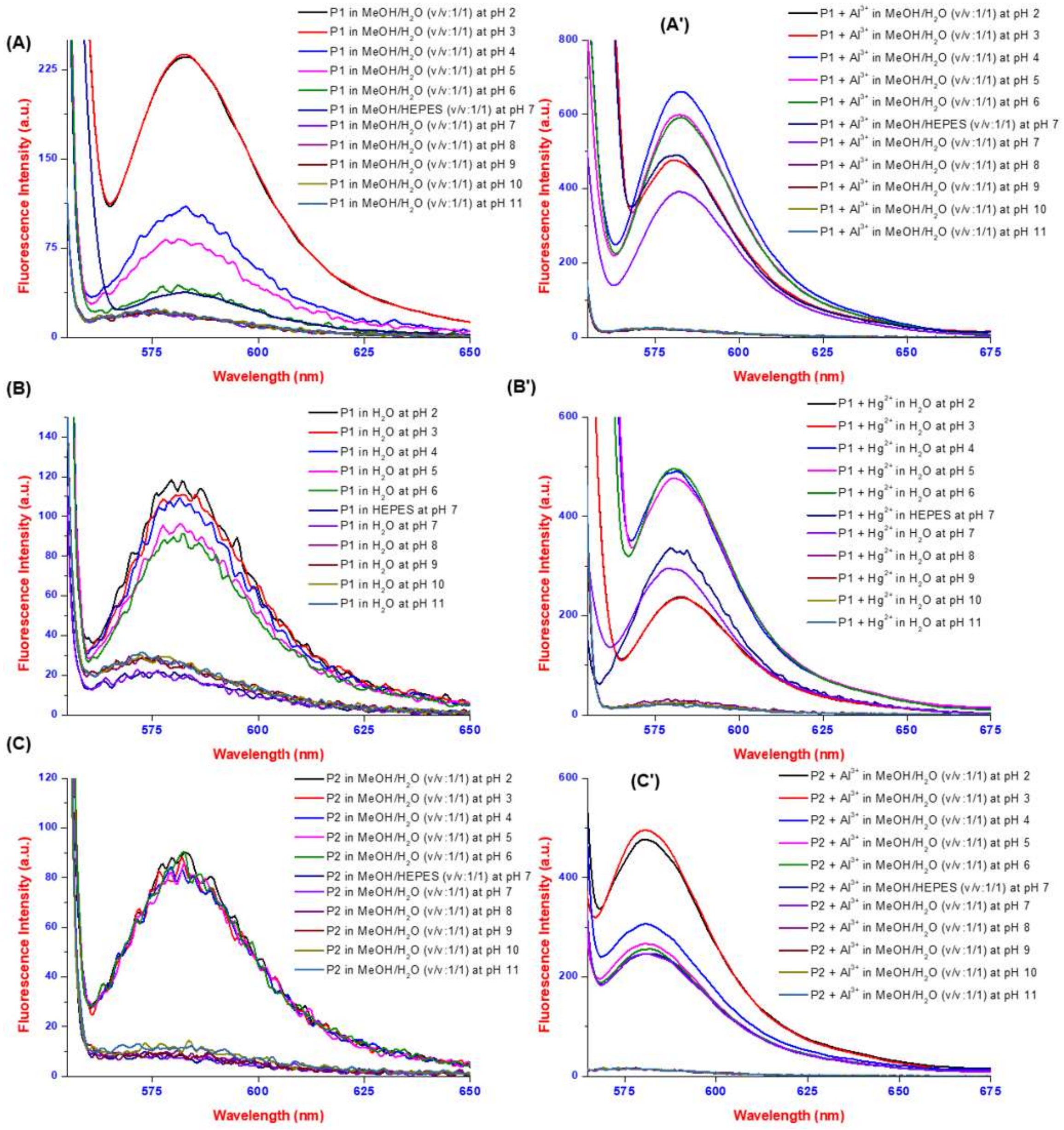

Figure S8. The fluorescence spectras of probes and probes (**A**-**C**) with [$AlCl_3$] / [$HgCl_2$] (**A'**-**C'**) at different pH (2–11) in selected solvent systems, the pH values were modulated by adding 75% HCl or NaOH solution.

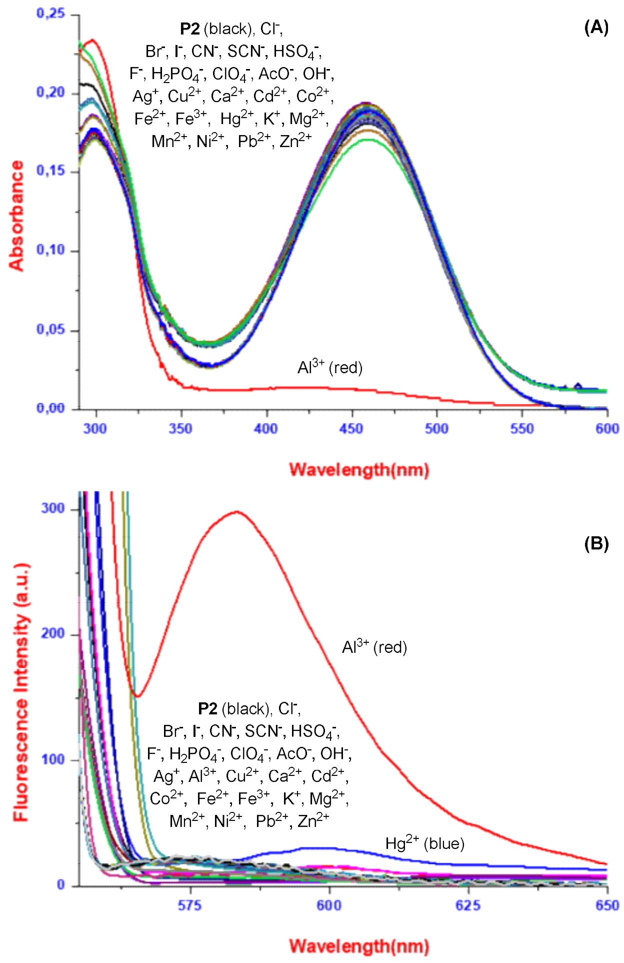

Figure S9. The UV-Vis (**A**) and fluorescence (**B**) spectras of **P2** with the absence and presence of ions in MeOH/H_2O

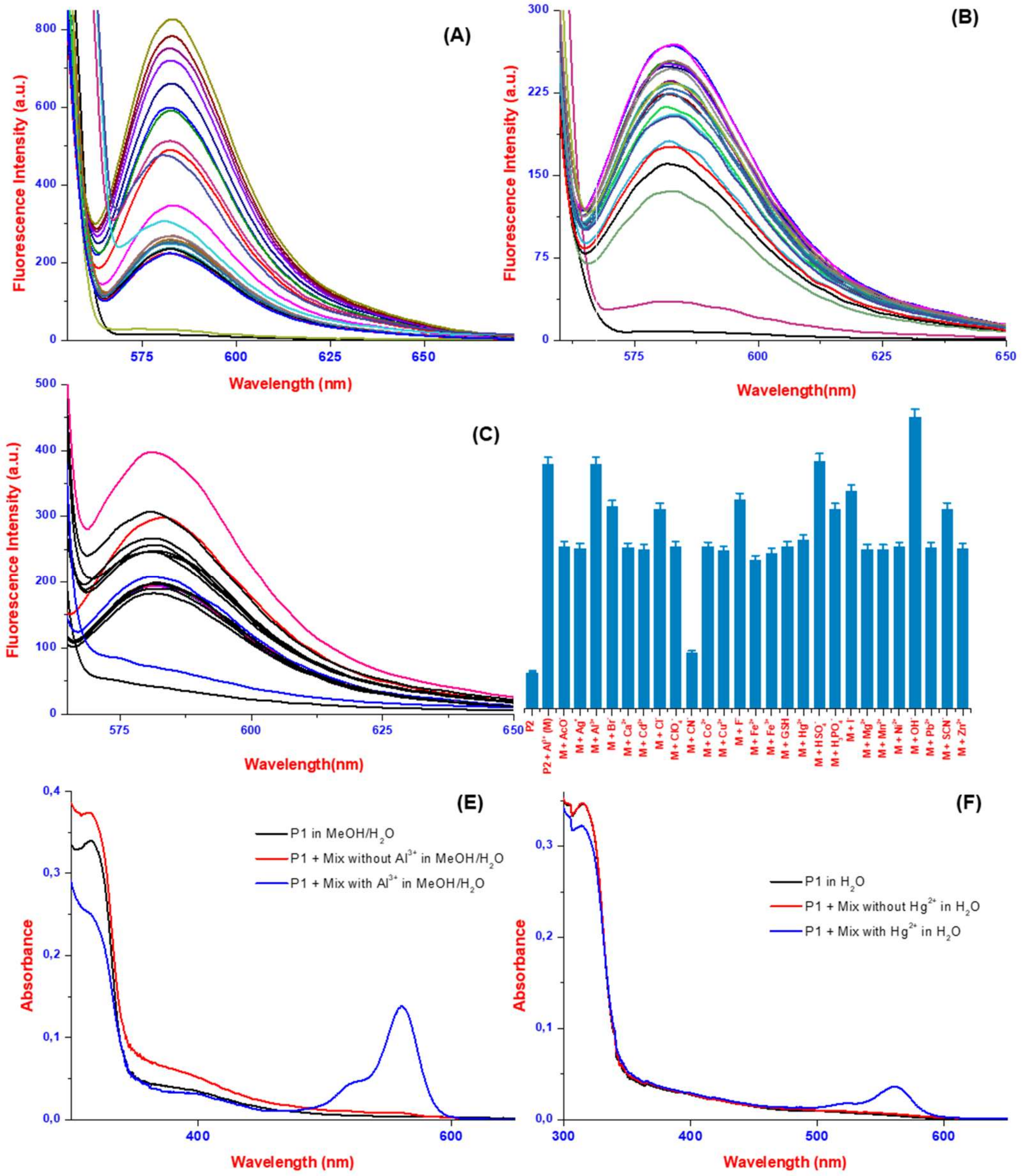

Figure S10. The fluorescence spectra of **P1**-Al^{3+} (**A**), **P1**-Hg^{2+} (**B**), **P2**-Al^{3+} (**C**) upon adding different ions up to 1 equivalent, and the UV-Vis spectras of the probe with the mixed cations in MeOH/H_2O (**E**) / in H_2O (**F**)

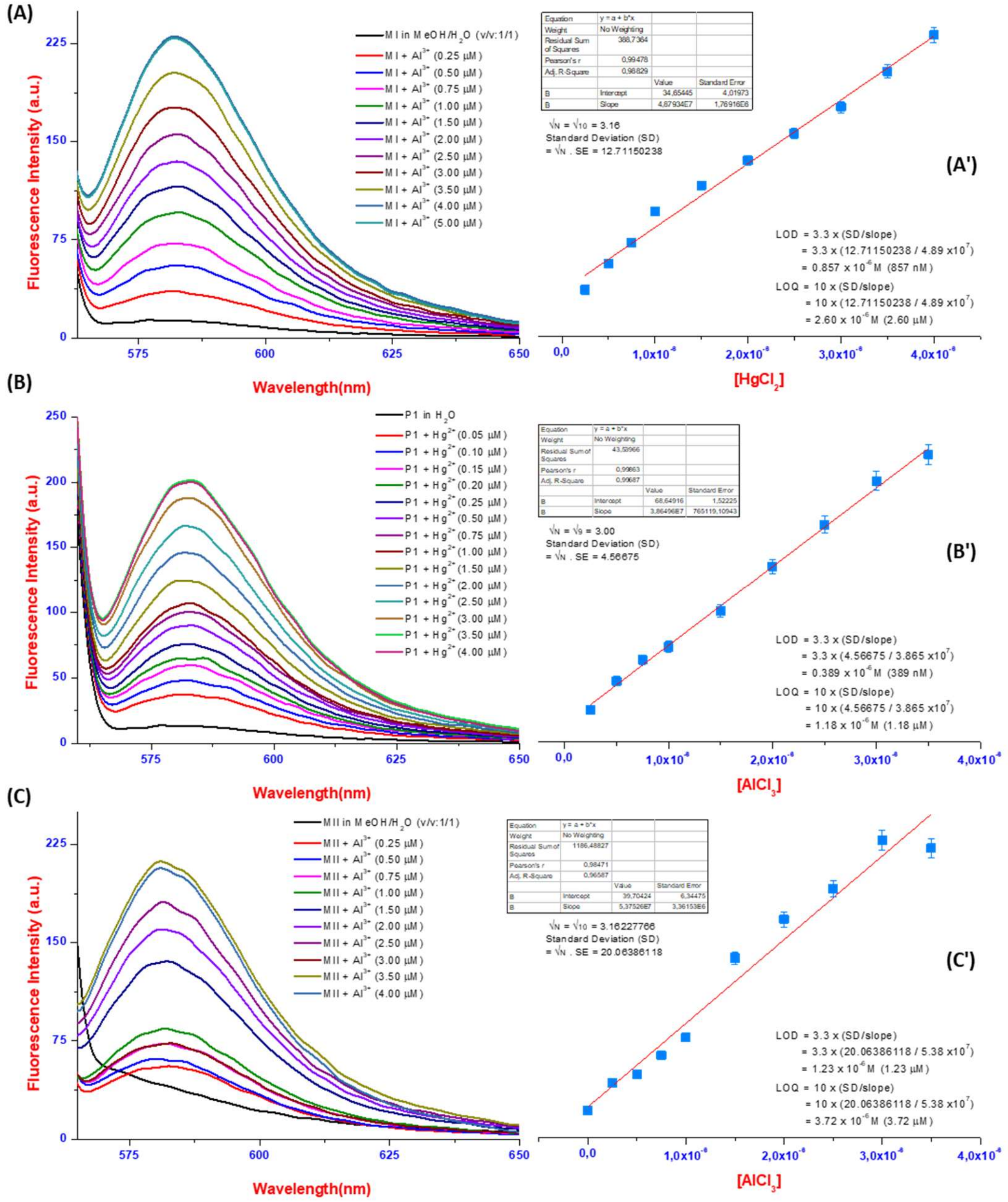

Figure S11. The fluorescence titration spectra of (**A**) **P1** with Al^{3+},(**B**) **P1** with Al^{3+}, (**C**) **P2** with Al^{3+} ions, and the change fluorescence intensity of **P1** and **P2** with the increasing concentration of Al^{3+} (**A'**), Hg^{2+} (**B'**), and Al^{3+} (**C'**).

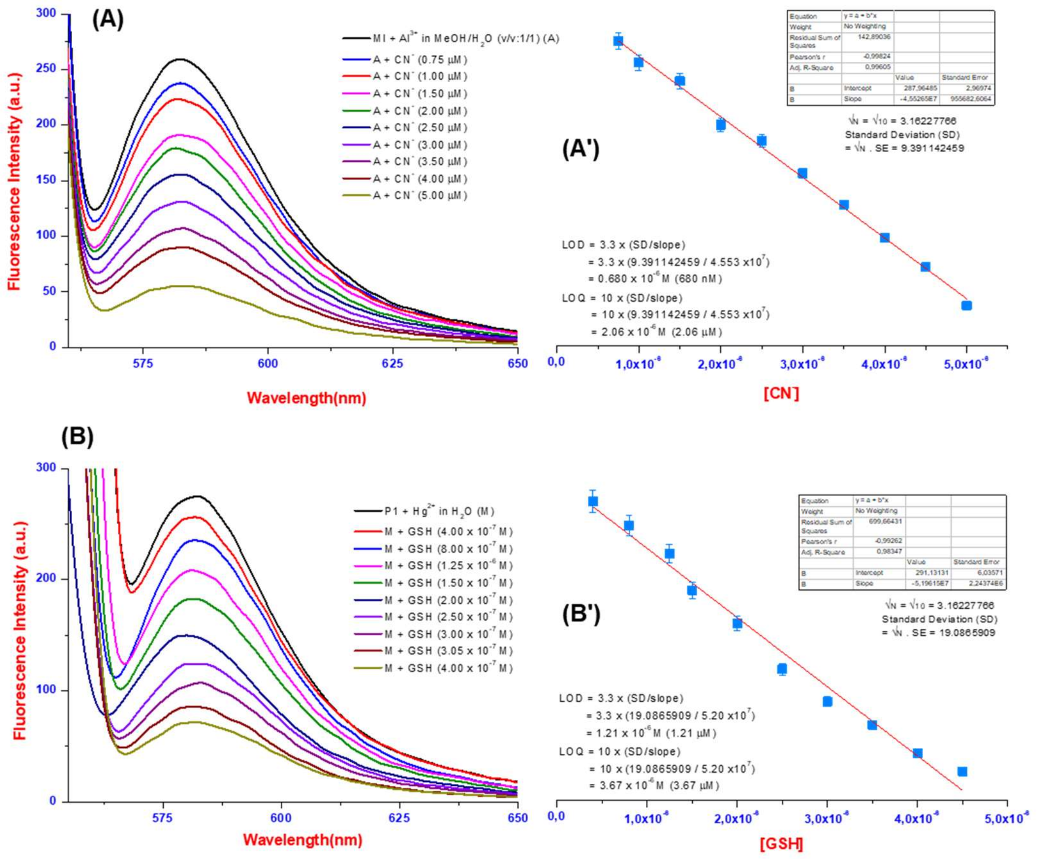

Figure S12. The fluorescence titration spectra of (**A**) **P1**/Al^{3+} with CN^-,(**B**) **P1**/Hg^{2+} with GSH, and the change in fluorescence intensity of **P1**/Al^{3+} and **P1**/Hg^{2+} with the increasing concentration of CN^- (**A'**), and GSH (**B'**)

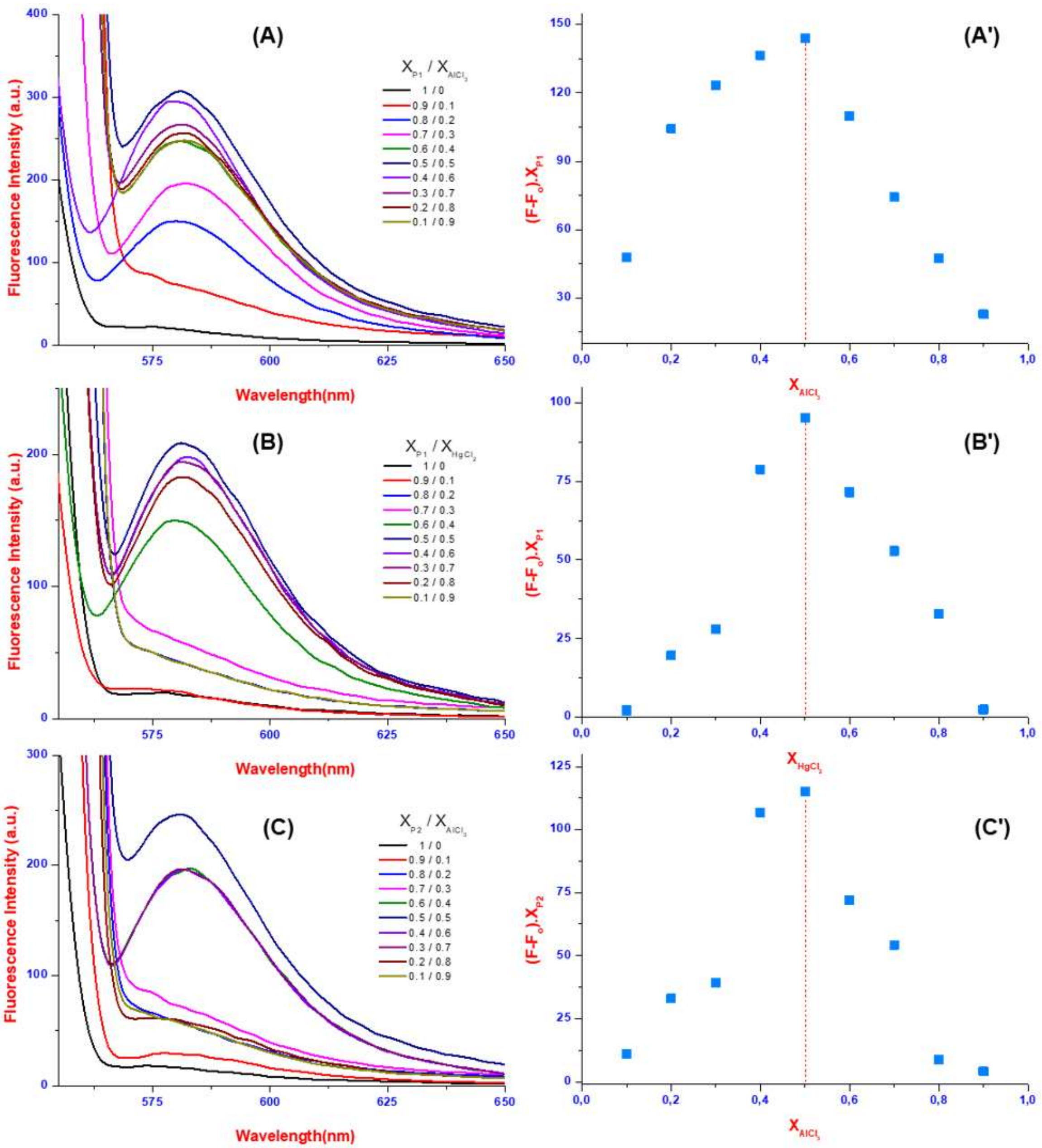

Figure S13. The Job plot fluorescence spectras of **P1**/$AlCl_3$ (**A** and **A'**), **P1**/$HgCl_2$ (**B** and **B'**), and **P2**/$AlCl_3$ (**C** and **C'**)

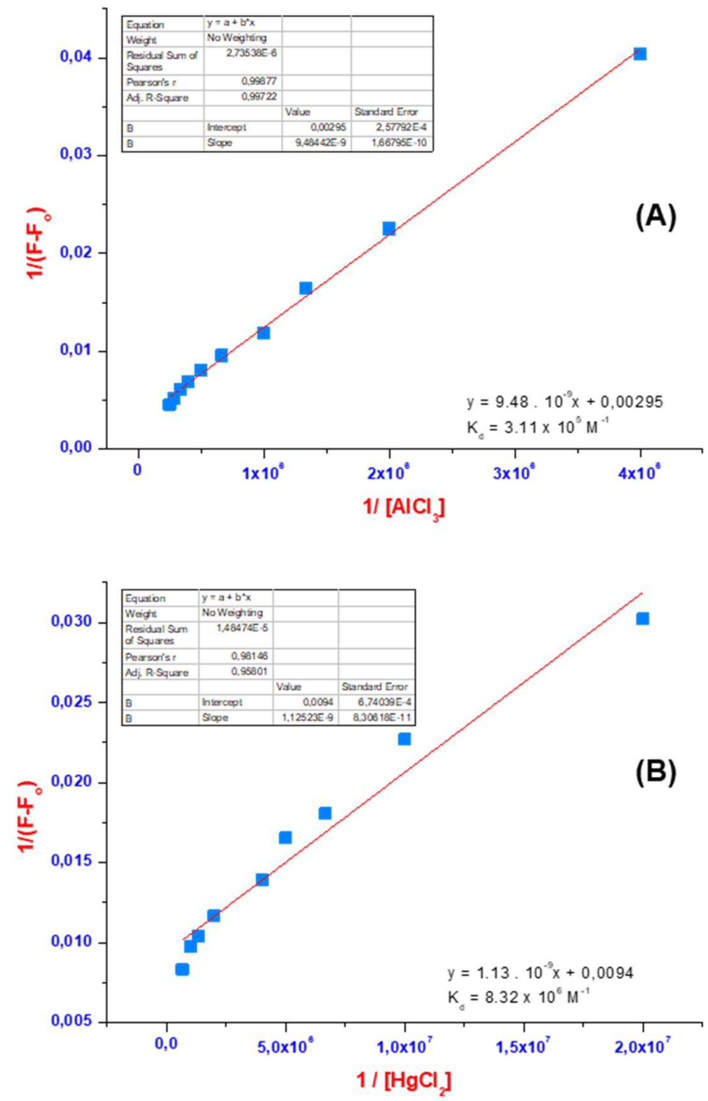

Figure S14. Benesi–Hildebrand plot based on a 1:1 association stoichiometry between **P1** with Al^{3+} (**A**), and Hg^{2+} (**B**) ions

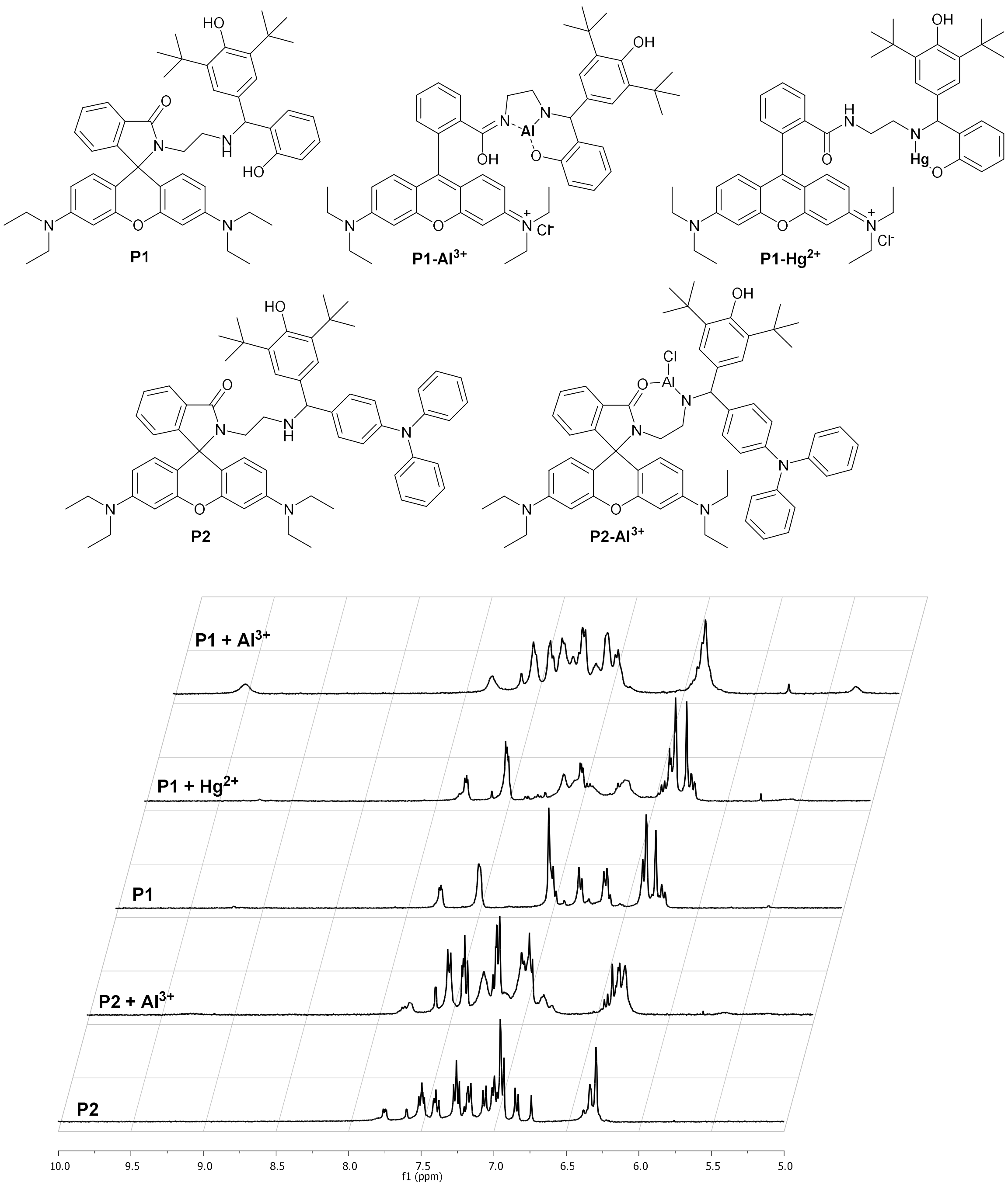

Figure S15. Change in partial ^{1}H-NMR (400 MHz) spectra of probes (**P1** and **P2**) with $AlCl_3$ and $HgCl_2$ in DMSO-d6.

P1+Al^{3+}: HRMS (ESI-TOF) m/z: $[M]^+$ calcd for $C_{51}H_{62}N_4O_4AlCl$, 856.4269; found 856.4277.

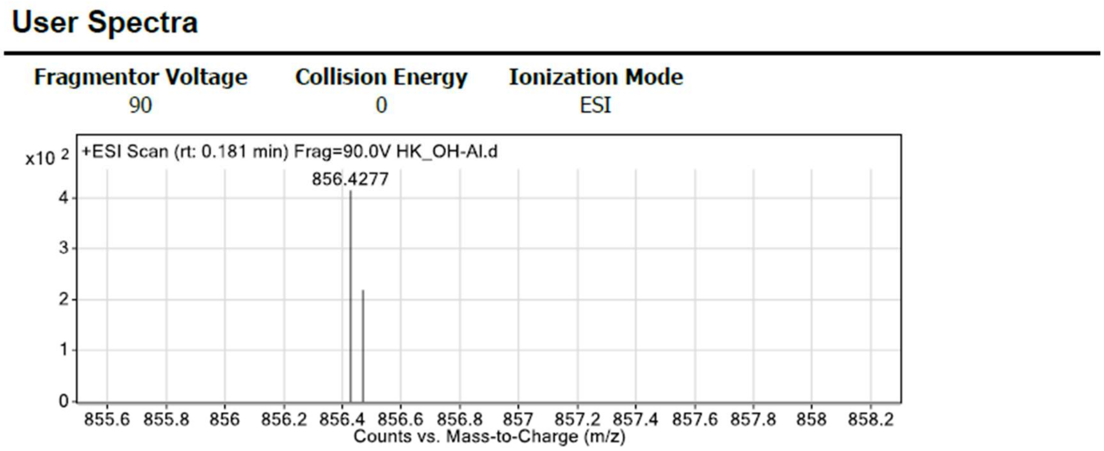

P1+Hg^{2+}: HRMS (ESI-TOF) m/z: $[M]^{+2}$ calcd for $C_{51}H_{63}N_4O_4HgCl$, 513.2093; found 513.2813.

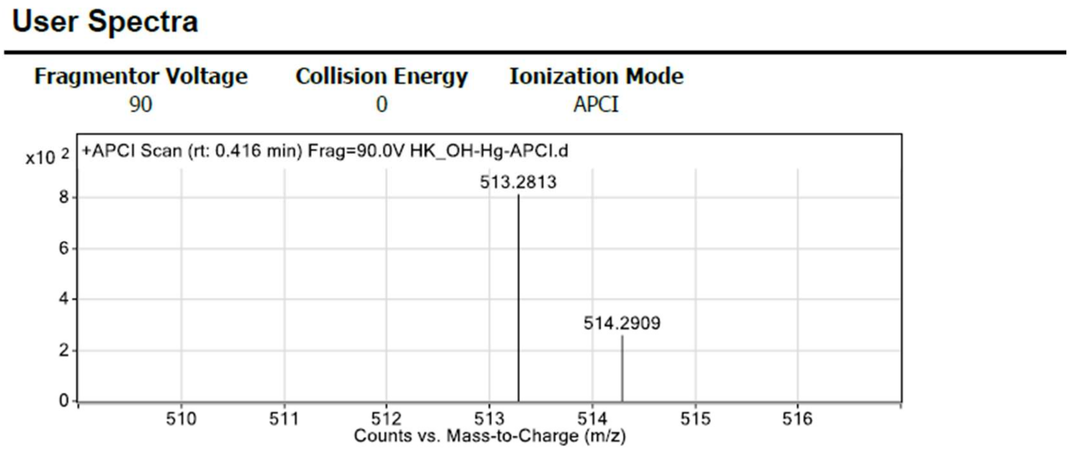

Figure S16. HRMS (ESI-TOF) spectras of P1+Al^{3+} (m/z = 1) and P1+Hg^{2+} (m/z = 2)

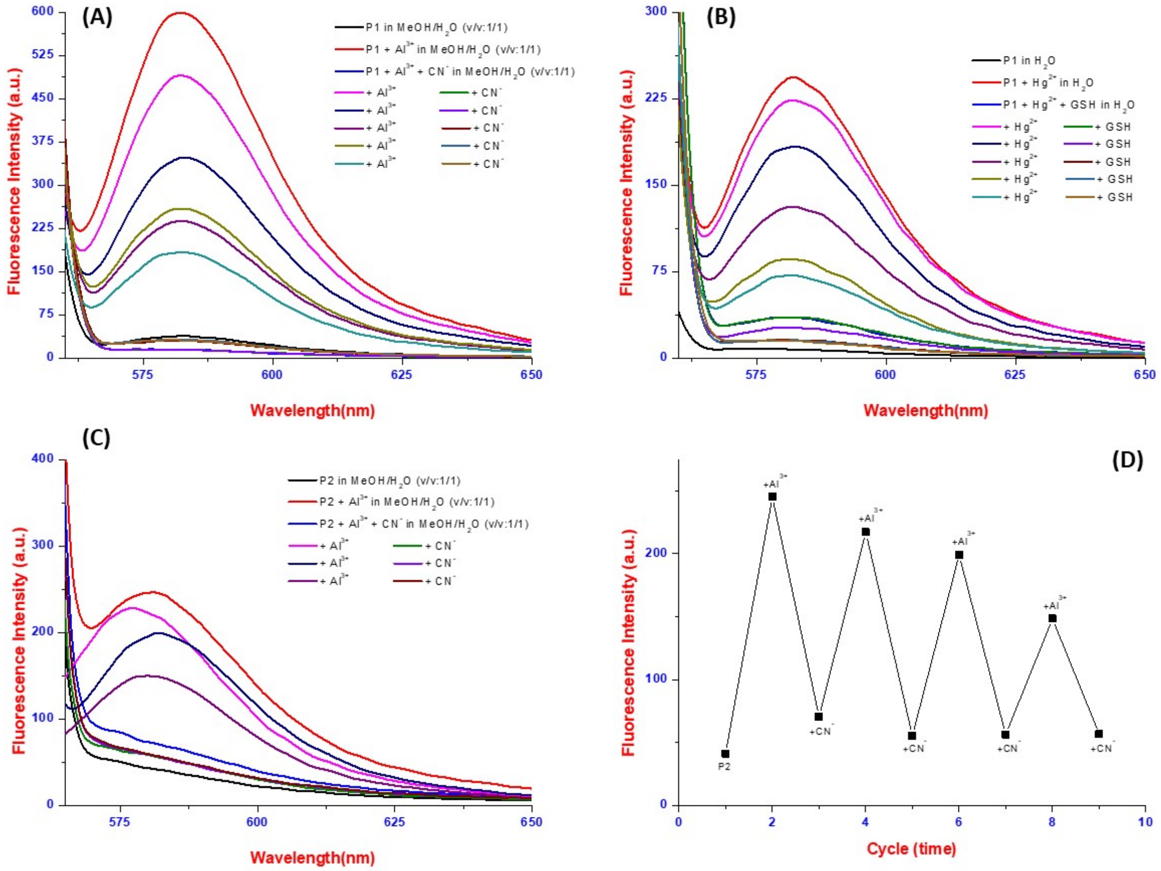

Figure S17. Reversible switching of the fluorescence spectrum of **P1** (**A** and **B**) and **P2** (**C**) with alternate addition of Al^{3+} / Hg^{2+} and CN^-/GSH, and (**D**) the "IMPLICATION" logic gate for **P2**+Al^{3+} with CN^-.

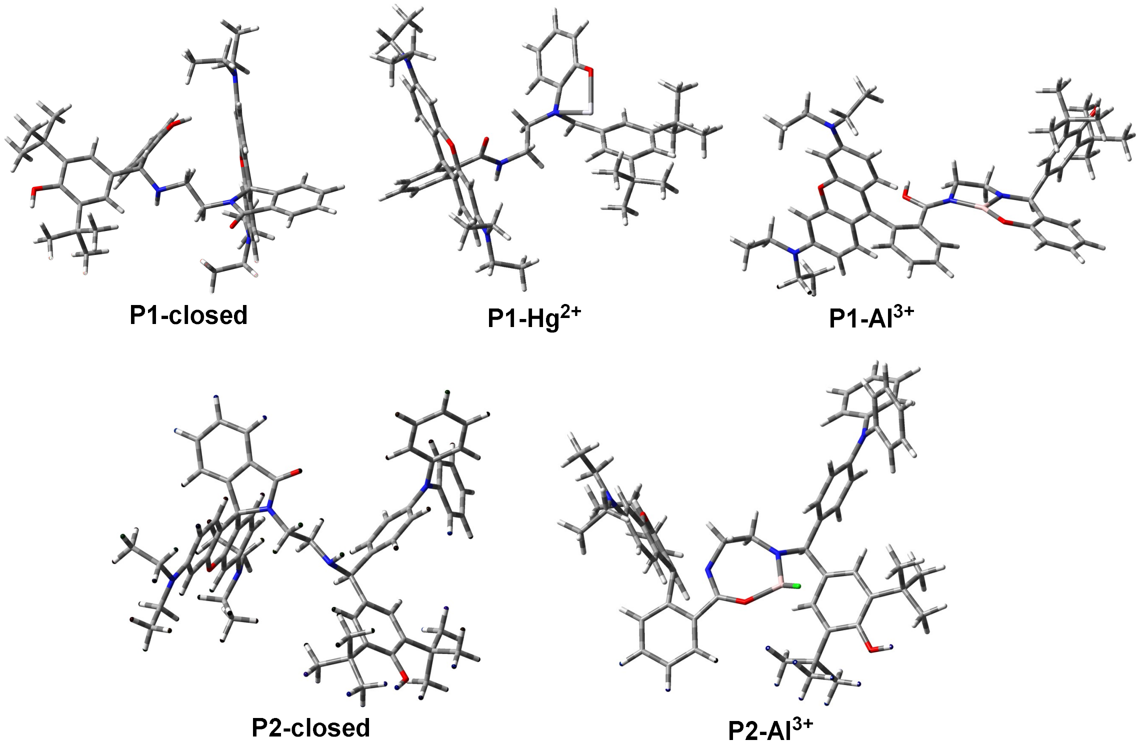

Figure S18. The optimized molecular geometries of probes **P1**/**P2**/**P1**-Hg^{2+}/**P1**-Al^{3+}/**P2**-Al^{3+}

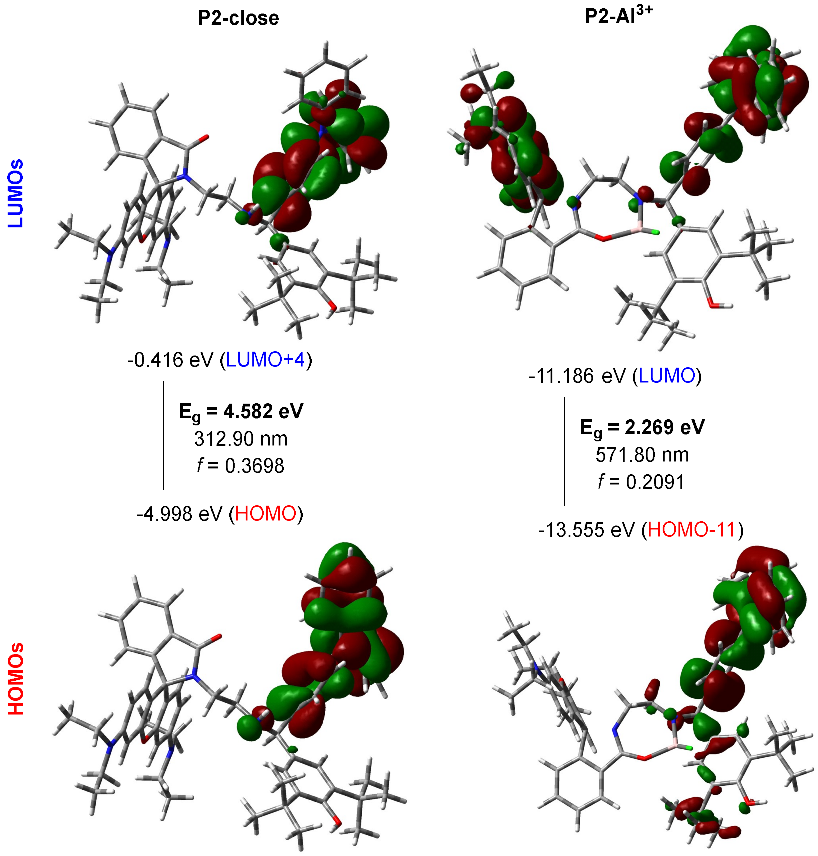

Figure S19. The HOMO/LUMO orbital distributions of **P2**-close and **P2**-Al^{3+}

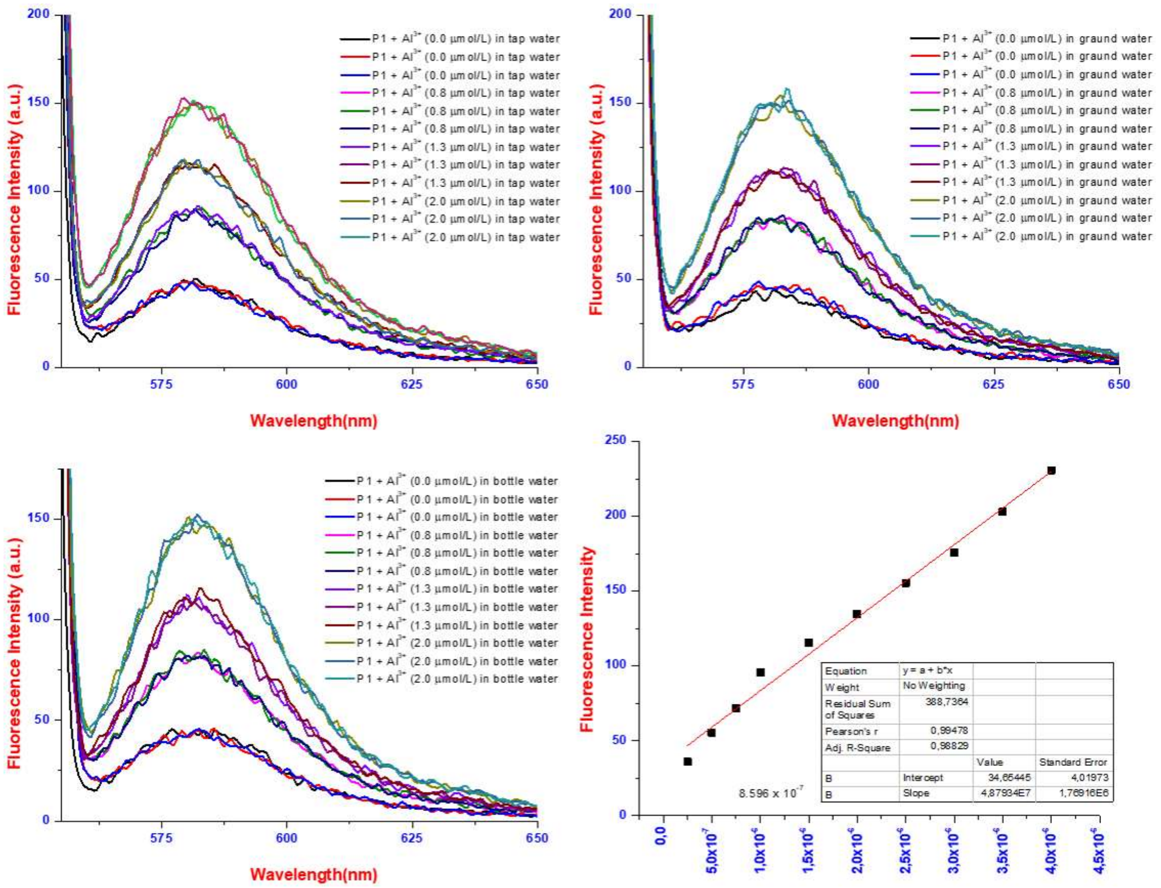

Figure S20. The fluorescence intensity of **P1** for the determination of Al^{3+} in real water samples

References

[1]. Kilic, H., Bozkurt, E. (2018). A rhodamine-based novel turn-on trivalent ions sensor. Journal of Photochemistry and Photobiology A: Chemistry, 363, 23-30.

[2]. Jarava-Barrera, C., Parra, A., López, A., Cruz-Acosta, F., Collado-Sanz, D., Cárdenas, D. J., Tortosa, M. (2016). Copper-catalyzed borylative aromatization of p-quinone methides: enantioselective synthesis of dibenzylic boronates. *ACS catalysis*, *6*(1), 442-446.
